# Large-scale analysis highlights obesity as a risk factor for chronic, non-communicable inflammatory diseases

**DOI:** 10.3389/fendo.2025.1516433

**Published:** 2025-02-03

**Authors:** Sadegh Mousavi, Katja Bieber, Henner Zirpel, Artem Vorobyev, Henning Olbrich, Cristian Papara, David A. De Luca, Diamant Thaci, Enno Schmidt, Gabriele Riemekasten, Peter Lamprecht, Matthias Laudes, Khalaf Kridin, Ralf J. Ludwig

**Affiliations:** ^1^ Lübeck Institute of Experimental Dermatology, University of Lübeck, Lübeck, Germany; ^2^ Institute and Comprehensive Centre for Inflammatory Medicine, University of Lübeck, Lübeck, Germany; ^3^ Department of Dermatology, University Hospital Schleswig-Holstein Lübeck, Lübeck, Germany; ^4^ Department of Rheumatology and Clinical Immunology, University Hospital Schleswig-Holstein Lübeck, Lübeck, Germany; ^5^ Institute of Diabetes and Clinical Metabolic Research, University of Kiel, Kiel, Germany; ^6^ Azrieli Faculty of Medicine, Bar-Ilan University, Safed, Israel; ^7^ Unit of Dermatology and Skin Research Laboratory, Galilee Medical Center, Nahariya, Israel

**Keywords:** obesity, inflammation, risk, cohort study, racial disparities, sex differences

## Abstract

**Background:**

Overweight and obesity are a global pandemic, contributing to death and disability-adjusted life-years. Obesity is a major factor in the onset of chronic inflammatory diseases (CIDs). Yet, several knowledge gaps remain: For several CIDs, inconsistent results have been reported, relating to their obesity-imposed risk, data on most rare CIDs remain unavailable, sex differences and racial disparities remain mostly unaddressed.

**Methods:**

A large-scale cohort study compared the risk of developing 46 CIDs in individuals with overweight/obesity (n=3,101,824) to an equal number of non-overweight/obese individuals. Propensity score matching optimized between-group comparability, and sensitivity analyses assessed study robustness.

**Results:**

The risk of developing any CID was 28.48% in overweight/obese individuals versus 17.55% in non-overweight/obese controls, with a hazard ratio (95%-confidence interval) of 1.52 (1.509-1.521, p<0.0001). This risk was consistent across all sensitivity, sex-, and race-stratified analyses. Overweight and obesity were associated with an increased risk for 24 of 46 CIDs in the primary analysis and all sensitivity analyses. For 12 diseases, increased risks were confirmed to one of the two sensitivity analyses, while for 10 diseases, results were discordant. No increased risk was observed for one disease. In sex-stratified analysis, overweight and obesity posed a more pronounced risk for four CIDs in female individuals. In race-stratified analysis, overweight and obesity were linked to a higher risk for seven CIDs in White individuals and to one CID in “Black or African American” individuals.

**Conclusion:**

Overweight and obesity increase the risk for the majority of CIDs in a sex- and race-specific manner.

## Introduction

Globally, overweight and obesity are a major health burden. Their prevalence is continuously rising, and an increased body-mass index is among the top five risk factors for death and disability-adjusted life-years (1). Thus, overweight/obesity are seen as a major global endemic (2). The increase of the prevalence of overweight/obesity has been paralleled by a similar rise in chronic, non-communicable inflammatory diseases (CID) (3–5). Like overweight and obesity, CIDs, e.g., atopic dermatitis, systemic lupus erythematosus (SLE), and inflammatory bowel disease, impose a major health burden with several unaddressed medical needs (6).

Landmark publications have highlighted the risk of obese patients to develop CIDs, for example in psoriasis, asthma, type 1 diabetes, and hidradenitis suppurativa (7–10). Supporting this notion, a Mendelian randomization study demonstrated that genetically predicted body mass index (BMI) is associated with an increased risk for 4 out of 15 autoimmune diseases, i.e., asthma, hypothyroidism, psoriasis, and type 1 diabetes (11). Similar observations were made for rheumatoid arthritis (12). In addition to the increased risk of CID, the severity of clinical disease tends to be greater in obese as opposed to non-obese patients (13–15). In line, reduction of body weight is beneficial in already established CID: In the early 1990s a study on the impact of fasting followed by one-year vegetarian diet on rheumatoid arthritis (RA) was evaluated in a small-scale controlled study (16). This positive effect of diet in RA was validated in subsequent studies (17). In psoriasis, weight loss more than doubled the number of patients reaching remission with weight-adjusted immunosuppressive treatment (18). Hence, body weight has been demonstrated to have an impact on CID risk, severity, and treatment outcomes for a limited number of CIDs.

Despite these insights, there are several knowledge gaps relating to the impact of overweight and obesity on the risk of CID: First, for several CIDs discrepant results relating to the risk of overweight and obesity for the subsequent CID manifestation were published. For example, in RA several studies reported an increased risk of RA among obese individuals, whilst almost the same number of studies found no such relation (13). Similarly, in atopic dermatitis (AD) in a total of 43 studies, an almost equal number of studies reported either an association of obesity with AD risk and/or severity, or no impact on AD. One study reported that obesity was associated with a decreased risk of AD. However, sample sizes were usually below 5,000 AD patients in each study, which may explain the discrepant results (19). Regarding inflammatory bowel disease, i.e., ulcerative colitis (UC) and Crohn’s disease (CD), the Nurses’ Health Study (NHS) II identified obesity as a risk factor for CD, but not UC (20). By contrast, no impact of BMI on the risk of either inflammatory bowel disease was noted in the Danish National Birth Cohort (9). For SLE, discrepant results were obtained in the U.S. NHS cohorts: Whilst obesity was significantly associated with SLE risk in NHS II, this finding was not seen in NHS I and not seen in the meta-analysis of the data from both studies (21). For COPD, an overrepresentation of obese individuals was reported (22). By contrast another study found that low BMI is a risk factor for COPD (23). Second, in most studies, sex- and race-specific risks of obesity on CID risk have only rarely been considered. Hence, there remains a significant knowledge gap on the sex-and race-specific impact of obesity on CID development. Third, for many rare CIDs no data relating to the risk of overweight & obesity are available. In detail: For pemphigus and bullous pemphigoid (BP), a slight association with obesity had been reported (24). Yet, data from case-control studies on pemphigus and BP are so far lacking. For granulomatosis with polyangiitis (GPA), eosinophilic granulomatosis with polyangiitis (EGPA), mucous membrane pemphigoid, immune thrombocytopenic purpura, autoimmune hemolytic anemia, pernicious anemia, autoimmune hepatitis, primary biliary cirrhosis, myasthenia gravis, and many others, the risk of obesity on disease-induction is unknown. The elegant study by Harpsøe and colleagues addressed the risk of CID in the Danish National Birth Cohort, including 75,008 women with long-term follow up. This setting is ideally suited to address the risk of common, but not rare, CIDs. For example, 315 RA cases were noted in the population, whilst for dermatitis herpetiformis, dermatomyositis, primary biliary cirrhosis and GPA 10 or fewer incident cases were recorded. Of note, for none of these later diseases, an impact of BMI on disease risk was noted (9).

Clarification of these discrepancies and filling of the aforementioned knowledge gaps is of importance for individual health and the healthcare system. We thus evaluated the impact of overweight and obesity using the data provided in the US Collaborative Network of TriNetX, which, at the time of analysis, provided access to electronic health records (EHRs) from over 100 million patients (25), on the risk of subsequent development of 46 CIDs across several medical fields. The TriNetX database was selected because of this high number of EHRs. Retrieving data from this resource ensures a sufficient number of outcomes also from rare diseases and in parallel allows subgroup analysis aiming to identify potential sex- or racial disparities relating to the obesity-imposed CID risk. Given that obesity is identified as a clinically relevant risk factor for CIDs, measures for prevention or treatment of obesity hold the potential to not only prevent the clinical manifestation of metabolic and cardiovascular diseases but also reduce the incidence of CIDs.

## Methods

### Study design and database

**Figure 1:**
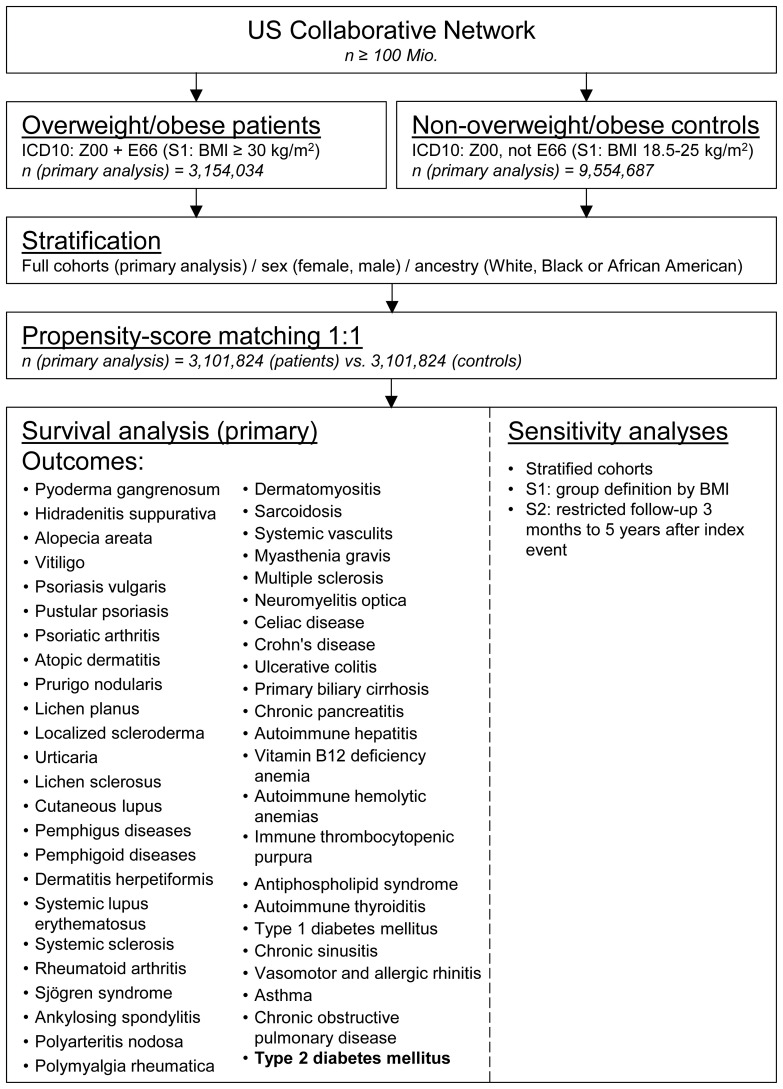
Study outline. Electronic health records were collected from the United States collaborative network of TriNetX. Eligible individuals were identified by a health-care encounter documents by ICD-10CM: Z00 and assigned to an obese patients’ or non-obese control cohort by registration of ICD-10CM: E66 (index event). After propensity-score matching, survival analyses considering chronic inflammatory diseases as well as type 2 diabetes mellitus were performed. For sensitivity analyses, age- or ancestry-defined stratification, cohort assignment by document body-mass index (S1), or shorter follow-up (S2) were employed.

A global population-based retrospective cohort study with propensity-score matching was performed following previously published protocols (26–28). In detail, the US Collaborative Network of TriNetX was used to identify EHRs with a diagnostic code for overweight and obesity and non-overweight/obese controls in adults. At the time of data retrieval and analysis, TriNetX provided access to over 120 million EHRs in five different Networks. The US Collaborative Network was chosen as it includes the second largest number of EHRs and the most detailed granular information for each EHR among the different networks provided by TriNetX (25, 29). Based on a collaboration between the University Clinic Schleswig Holstein (UKSH) and TriNetX, UKSH researchers have access to the TriNetX database and analytical tools. In EHRs with a diagnostic code for overweight and obesity and non-overweight/obese controls the risk to develop chronic, non-communicable inflammatory diseases (n=46) was assessed. As a control, the risk to develop type two diabetes mellitus (T2DM) was contrasted among the groups. To allow for a better comparability among groups propensity-score-matching (PSM) was used. The study’s robustness was assessed through two sensitivity analyses: In the first sensitivity analysis (S1), the BMI was utilized to define overweight/obesity. EHRs indicating a BMI of 18.5 to 25 kilograms per square meter (kg/m^2^) were categorized as normal weight controls, while those with a BMI of 30 kg/m^2^ or more were classified as overweight/obese. In the second sensitivity analysis (S2), only outcomes occurring from three months to five years after the index event were considered. Lastly, to identify potential sex-specific effects or racial disparities, the CID risk was assessed in EHRs stratified for sex (female and male) and race (“Black or African American” and White). Outcomes were defined prior to data acquisition and analyzed after propensity-score matching. The study design is depicted in **Figure 1**.

### Ethics statement

The data reviewed is a secondary analysis of existing data, does not involve intervention or interaction with human subjects, and is de-identified per the de-identification standard defined in Section §164.514(a) of the HIPAA Privacy Rule. The process by which the data is de-identified is attested to through a formal determination by a qualified expert as defined in Section §164.514(b) (1) of the HIPAA Privacy Rule. This formal determination by a qualified expert refreshed on December 2020. Thus, our study did not require Institutional Review Board approval.

### Study population and definition of eligible patients

For all analyses, EHRs with the code ICD10:Z00, coding for “Encounter for general examination without complaint, suspected or reported diagnosis” and with an age of 18 years or older were retrieved from the US Collaborative Network of TriNetX. For the primary analysis, the overweight/obese cohort had to have the additional code of “overweight and obesity” (ICD10:E66). For the non- overweight/obese ICD10:E66 served as an exclusion criterion. Documentation of overweight/obesity or health care encounter defined the index event. For sensitivity analysis S1, all EHRs were also retrieved from those with documented ICD10:Z00. Within this cohort, a BMI (LOINC 391 56-6)≥30 kg/m^2^ defined the overweight/obese group, and a BMI ranging from 18.5 to 25.0 kg/m^2^ defined the non-obese/overweight group.

### Follow-up and definition of outcomes

In the primary analysis, as well as in sensitivity analysis S1, the time for follow-up was unrestricted. By contrast, in sensitivity analysis S2, outcomes occurring from three months to five years after the respective index events were considered. Given that overweight/obesity is a well-known risk factor for the development of T2DM (30), we utilized T2DM as an outcome to validate our analytical pipeline. The primary endpoint for all analyses was the occurrence of any of the CIDs listed in **Supplementary Table 1**. Outcomes prior to the index events were excluded from analysis.

### Covariates

To allow a better comparability among the groups PSM for several covariates, including demographic factors and potential risk factors for chronic, non-communicable inflammatory diseases, was used. PSM is a method utilized in observational studies to balance cohorts by matching individuals based on their likelihood (propensity) of being assigned to a particular treatment or exposure group. This aims to mitigate the influence of potential confounding variables, thus enhancing the comparability of the different groups. Several previous studies employed PSM to reduce potential biases inherent in observational research. PSM is potentially advantageous over conventional covariate adjustment methods (31). To account for potential risk factors confounding to the risk for chronic, non-communicable inflammatory diseases, the following variables were included in the PSM: Age at index (years, continuous), female sex (binary), White race (binary), personal history of nicotine dependence (ICD10:Z87.891, binary), nicotine dependence (ICD10:F17, binary), reaction to severe stress, and adjustment disorders (ICD10:F43, binary), and problems related to life management difficulty (ICD10: Z73, binary). The matrix row order was randomized after data retrieval. A propensity-score for each patient was generated by logistic regression and matching was performed 1:1 using the greedy nearest neighbor approach with a caliper distance of 0.1 using the software package scikit-learn in Python (https://www.jmlr.org/papers/volume12/pedregosa11a/pedregosa11a.pdf?ref=https). Baseline characteristics were re-evaluated and reported after matching, differences were compared by t-test for continuous and z-test for binary or categorical variables.

### Statistical analysis

The statistical analysis was performed using the Survival package v3.2-3 in R (R Foundation for Statistical Computing, Vienna, Austria) and validated by comparison with the outputs of SAS version 9.4 (SAS, Cary, NC); graphs were created using GraphPad Prism (GraphPad Software Inc., Boston, MA. Relative risks and risk differences were calculated. Survival analyses were performed using the Kaplan-Meier method (KM). KM-curves were compared using the Log-rank test. Hazard ratio and its associated confidence intervals, together with the test for proportionality, are calculated using R’s Survival package v3.2-3, whereby large Chi-square values are indicative of low proportionality, whereas small Chi-square values suggest higher proportionality. If Chi-square values were greater or equal to 10, odds ratio is additionally indicated. For the analysis of the primary endpoint (occurrence of any CID) a p value of 0.05 was considered significant. To counter the bias of multiple testing when investigating the risk to develop any of the 46 CIDs Bonferroni correction was applied (α = 0.05/46 = 0.0011).

## Results

### Cohort description and the risk of type 2 diabetes development in overweight/obese individuals

In the primary analysis, 3,154,034 electronic health records (EHRs) were identified with a diagnostic code indicating overweight/obesity (cases), while 9,554,687 EHRs without this diagnostic code were retrieved (controls). Prior to PSM, substantial disparities were observed in demographics and potential risk factors for CIDs. For instance, individuals categorized as cases had a mean (SD) age of 47.8 ± 18.7 years, whereas controls exhibited a notably younger mean (SD) age of 43.4 ± 21.2 years (p<0.0001, standardized difference (std. diff.) 0.2175). Moreover, the prevalence of nicotine dependence was higher among cases (9.4%) compared to controls (4.6%, p<0.0001, std. diff. 0.1882). Similar differences were consistent across all sensitivity analyses, as well as in analyses stratified by sex or race/ancestry. After PSM for all seven covariates, a total of 3,101,824 EHRs were retrieved for each of the two groups in the primary analysis. Furthermore, differences in demographics and putative risk factors for chronic, non-communicable inflammatory diseases were lessened. For example, the mean (SD) age was 47.7 ± 18.7 years in cases and 47.8 ± 18.8 years in controls (p<0.0001, std. diff. 0.0048). Likewise, prevalence of nicotine dependence amounted to 9.05% in overweight/obese individuals, and 9.17% in non- overweight/obese control (p<0.0001, std. diff. 0.0041). **Table 1** details all data pertaining to the description of cohorts.

**Table 1:** Description of cohorts.

Analysis	Characteristic	Before PSM				After PSM			
		Overweight and obesity (Cases)	Controls	P Value	Std diff.	Overweight and (Cases)	Controls	P Value	Std diff.
Primary	Number of participants (n)	3,154,034	9,554,687	-	-	3,101,824	3,101,824	-	-
	Age at index (years, Mean ± SD)	47.8 ± 18.7	43.4 ± 21.2	< 0.0001	0.2175	47.7 ± 18.7	47.8 ± 18.8	< 0.0001	0.0048
	White (%)	62.07	60.158	< 0.0001	0.0392	62.013	62.053	0.3140	0.0008
	Female (%)	57.755	51.787	< 0.0001	0.1201	57.684	57.444	< 0.0001	0.0049
	Personal history of nicotine dependence (%)	9.25	3.501	< 0.0001	0.2370	8.919	8.844	0.0009	0.0027
	Nicotine dependence (%)	9.352	4.583	< 0.0001	0.1882	9.048	9.165	< 0.0001	0.0041
	Reaction to severe stress, and adjustment disorders (%)	4.278	1.808	< 0.0001	0.1442	4.092	4.198	< 0.0001	0.0053
	Problems related to life management difficulty (%)	0.806	0.191	< 0.0001	0.0874	0.641	0.538	< 0.0001	0.0134
S1	Number of participants (n)	1,526,479	1,340,976	-	-	1,073,847	1,073,847	-	-
	Age at index (years, Mean ± SD)	45.5 ± 17.9	40.6 ± 22.8	< 0.0001	0.2408	44 ± 19.9	43.9 ± 20.9	0.0585	0.0026
	White (%)	65.524	65.474	< 0.0001	0.0010	65.762	64.132	< 0.0001	0.0342
	Female (%)	59.459	60.337	< 0.0001	0.0179	63.762	60.414	< 0.0001	0.0690
	Personal history of nicotine dependence (%)	6.229	5.649	< 0.0001	0.0245	6.636	6.33	< 0.0001	0.0124
	Nicotine dependence (%)	6.703	5.789	< 0.0001	0.0378	6.92	6.511	< 0.0001	0.0163
	Reaction to severe stress, and adjustment disorders (%)	3.023	2.361	< 0.0001	0.0409	3.394	2.393	< 0.0001	0.0598
	Problems related to life management difficulty (%)	0.63	0.671	< 0.0001	0.0050	0.753	0.673	< 0.0001	0.0095
S2	Number of participants (n)	3,279,943	9,836,698	-	-	3,227,331	3,227,331	-	-
	Age at index (years, Mean ± SD)	47.9 ± 18.7	43.5 ± 21.3	< 0.0001	0.2205	47.9 ± 18.7	48 ± 18.8	< 0.0001	0.0031
	White (%)	63.344	61.079	< 0.0001	0.0470	63.288	63.311	0.5587	0.0470
	Female (%)	57.681	51.856	< 0.0001	0.1163	57.614	57.337	< 0.0001	0.0053
	Personal history of nicotine dependence (%)	9.344	3.586	< 0.0001	0.2354	9.029	8.987	0.0638	0.0019
	Nicotine dependence (%)	9.337	4.643	< 0.0001	0.1844	9.05	9.178	< 0.0001	0.0048
	Reaction to severe stress, and adjustment disorders (%)	4.371	1.861	< 0.0001	0.1446	4.185	4.287	< 0.0001	0.0056
	Problems related to life management difficulty (%)	0.785	0.188	< 0.0001	0.0859	0.624	0.525	< 0.0001	0.0134
Female	Number of participants (n)	1,896,787	5,105,814	-	-	1,855,973	1,855,973	-	-
	Age at index (years, Mean ± SD)	46.9 ± 18.6	43.4 ± 21.2	< 0.0001	0.1642	46.9 ± 18.6	47 ± 18.7	< 0.0001	0.0060
	White (%)	64.236	64.645	< 0.0001	0.0032	64.169	64.26	0.0660	0.0025
	Female (%)	100	100	-	-	100	100	-	-
	Personal history of nicotine dependence (%)	8.257	3.136	< 0.0001	0.2150	7.721	7.502	< 0.0001	0.0058
	Nicotine dependence (%)	8.754	3.968	< 0.0001	0.1933	8.211	8.469	< 0.0001	0.0075
	Reaction to severe stress, and adjustment disorders (%)	5.256	2.229	< 0.0001	0.1616	4.939	5.014	0.0009	0.0018
	Problems related to life management difficulty (%)	0.882	0.223	< 0.0001	0.0915	0.7	0.574	< 0.0001	0.0142
Male	Number of participants (n)	1,218,271	4,219,865	-	-	1,203,562	1,203,562	-	-
	Age at index (years, Mean ± SD)	48.8 ± 18.9	42.8 ± 21.2	< 0.0001	0.2960	48.7 ± 18.9	48.7 ± 18.9	0.4324	0.0010
	White (%)	70.526	64.142	< 0.0001	0.1364	70.487	70.402	0.1453	0.0019
	Female (%)	0	0	-	-	0	0	-	-
	Personal history of nicotine dependence (%)	10.864	4.045	< 0.0001	0.2618	10.692	10.701	0.8283	0.0003
	Nicotine dependence (%)	10.365	5.393	< 0.0001	0.1854	10.211	10.239	0.4721	0.0009
	Reaction to severe stress, and adjustment disorders (%)	3.131	1.468	< 0.0001	0.1111	3.065	3.145	0.0003	0.0046
	Problems related to life management difficulty (%)	0.734	0.167	< 0.0001	0.0848	0.574	0.498	< 0.0001	0.0103
African American or Black	Number of participants (n)	602,124	1,194,648	-	-	582,484	582,484	-	-
	Age at index (years, Mean ± SD)	43.7 ± 18.5	39.7 ± 20.9	< 0.0001	0.1985	43.5 ± 18.5	43.7 ± 18.7	< 0.0001	0.0121
	White (%)	0	0	-	-	0	0	-	-
	Female (%)	70.1	53.898	< 0.0001	0.3386	69.699	69.377	0.0002	0.0070
	Personal history of nicotine dependence (%)	6.287	3.063	< 0.0001	0.1532	5.341	5.461	0.0040	0.0053
	Nicotine dependence (%)	9.195	6.298	< 0.0001	0.1085	8.717	8.827	0.0355	0.0039
	Reaction to severe stress, and adjustment disorders (%)	3.759	1.875	< 0.0001	0.1141	3.139	3.151	0.7062	0.0007
	Problems related to life management difficulty (%)	0.567	0.165	< 0.0001	0.0665	0.382	0.31	< 0.0001	0.0122
White	Number of participants (n)	2,075,874	5,994,544	-	-	2,042,756	2,042,756	-	-
	Age at index (years, Mean ± SD)	50 ± 18.2	45.3 ± 21.3	< 0.0001	0.2351	49.9 ± 18.2	50 ± 18.3	< 0.0001	0.0049
	White (%)	100	100	-	-	100	100	-	-
	Female (%)	58.492	54.883	< 0.0001	0.0729	58.455	58.277	0.0003	0.0036
	Personal history of nicotine dependence (%)	10.77	4.203	< 0.0001	0.2515	10.384	10.304	0.0078	0.0026
	Nicotine dependence (%)	10.15	5.126	< 0.0001	0.1900	9.793	9.838	0.1319	0.0015
	Reaction to severe stress, and adjustment disorders (%)	4.75	2.115	< 0.0001	0.1451	4.543	4.667	< 0.0001	0.0060
	Problems related to life management difficulty (%)	0.967	0.236	< 0.0001	0.0948	0.754	0.637	< 0.0001	0.0141

Data was retrieved from March 1^st^ to 8^th^, 2024.

Std diff., standardized difference.

**Figure 2:**
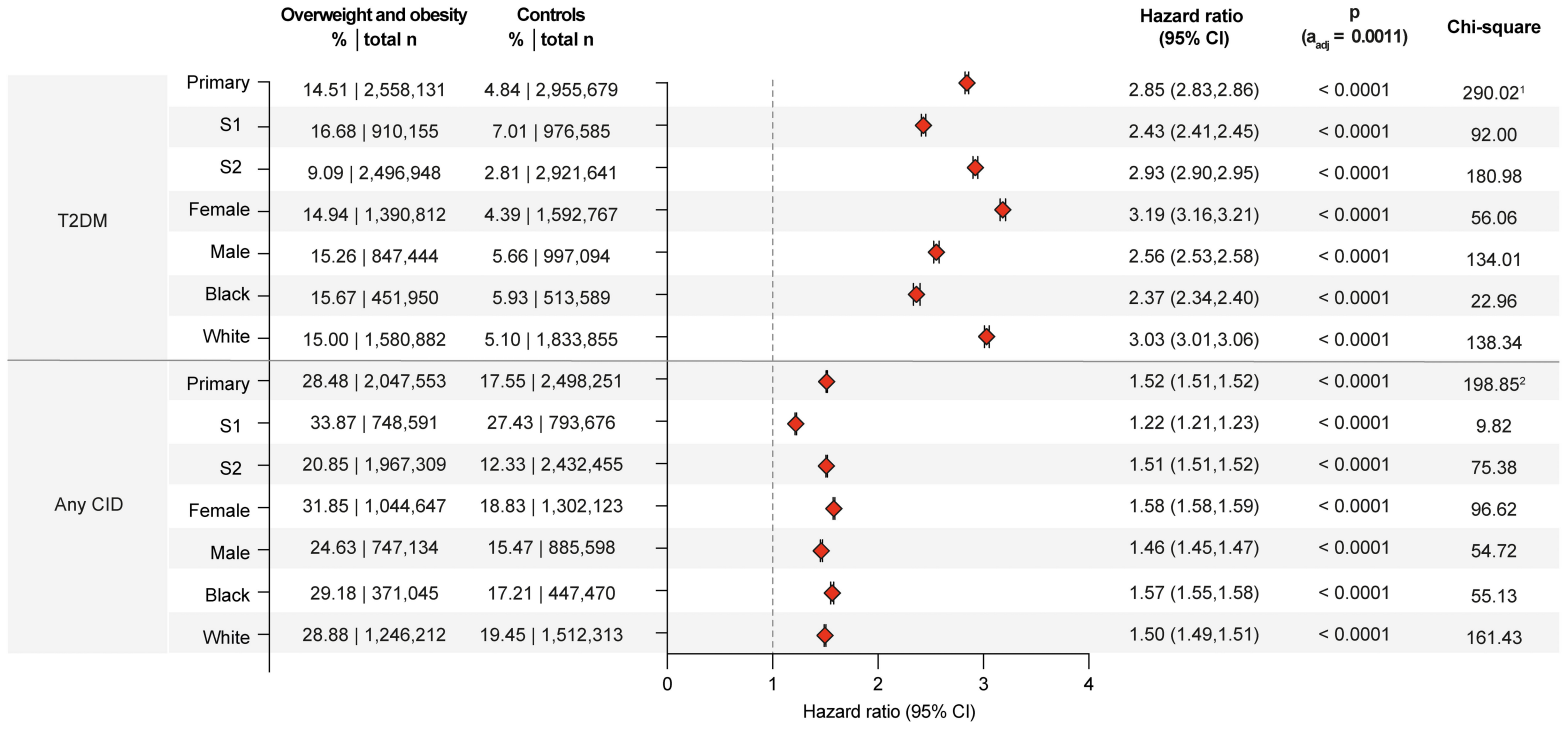
Overweight or obesity confer an increased risk for chronic, non-communicable inflammatory diseases. Risk of Type 2 diabetes mellitus (T2DM) and any of the investigated chronic, non-communicable inflammatory diseases (CID) in overweight and obese cases compared to non- overweight and obese controls. For comparisons with a highly violated proportionality assumption in the primary analysis, the odds ratio (OR) that also excludes outcomes prior to the index event, its’ 95%-confidence interval and p-value are provided in the footnotes: ^1^OR 3.338 (3.317,3.359) p<0.0001, ^2^OR 1.871 (1.863,1.879) p<0.0001. Please note that numbering of footnotes is based on **Supplementary Table 2**.

To weight the impact of overweight/obesity on subsequent CID manifestation and to validate the analytical pipeline, the well-established risk of overweight/obesity for the development of T2DM was determined (32). As detailed in **Supplementary Table 2** the risk for T2DM amounted to 14.51% in overweight/obese individuals as opposed to 4.84% in non- overweight/obese controls. This increased risk for T2DM in overweight/obese individuals translated in a HR of 2.86 (CI 2.83-2.86, p<0.0001). This finding remained consistent across all sensitivity analyses, as well as in all sex- and race-stratified analyses (**Figure 2**). This observation is also consistent with previous reports (32, 33), validates the analytical pipeline, and provides a comparison for the CID risk associated with overweight/obesity.

### Globally overweight/obesity increases the risk of subsequent CID

The predefined primary endpoint of the study was the occurrence of any CID following the index event. In the primary analysis, 28.48% of overweight/obese individuals experienced a documented instance of CID post-index event. In contrast, individuals without documented overweight/obesity had a CID risk of 17.55%. This difference corresponds to a HR of 1.52 (95% CI 1.51-1.52, p<0.0001). The increased CID risk persisted across both sensitivity analyses and all sex- and ancestry-stratified analyses. Specifically, the CID risk was consistent among female, male, Black or African American, and White individuals (**Figure 2**).

### Overweight and obesity is a risk factor for 24 of 46 investigated CIDs

At the single disease level, of the 46 investigated CIDs overweight/obesity was associated with an increased risk for 24 conditions. While some associations between obesity and these diseases are well-documented, many remain controversial or unexplored. Additionally, sex and racial differences in the association between obesity CIDs are understudied. These following passages will focus on these knowledge gaps, with all data provided in **Supplementary Table 2**.

#### Dermatological CIDs

**Figure 3:**
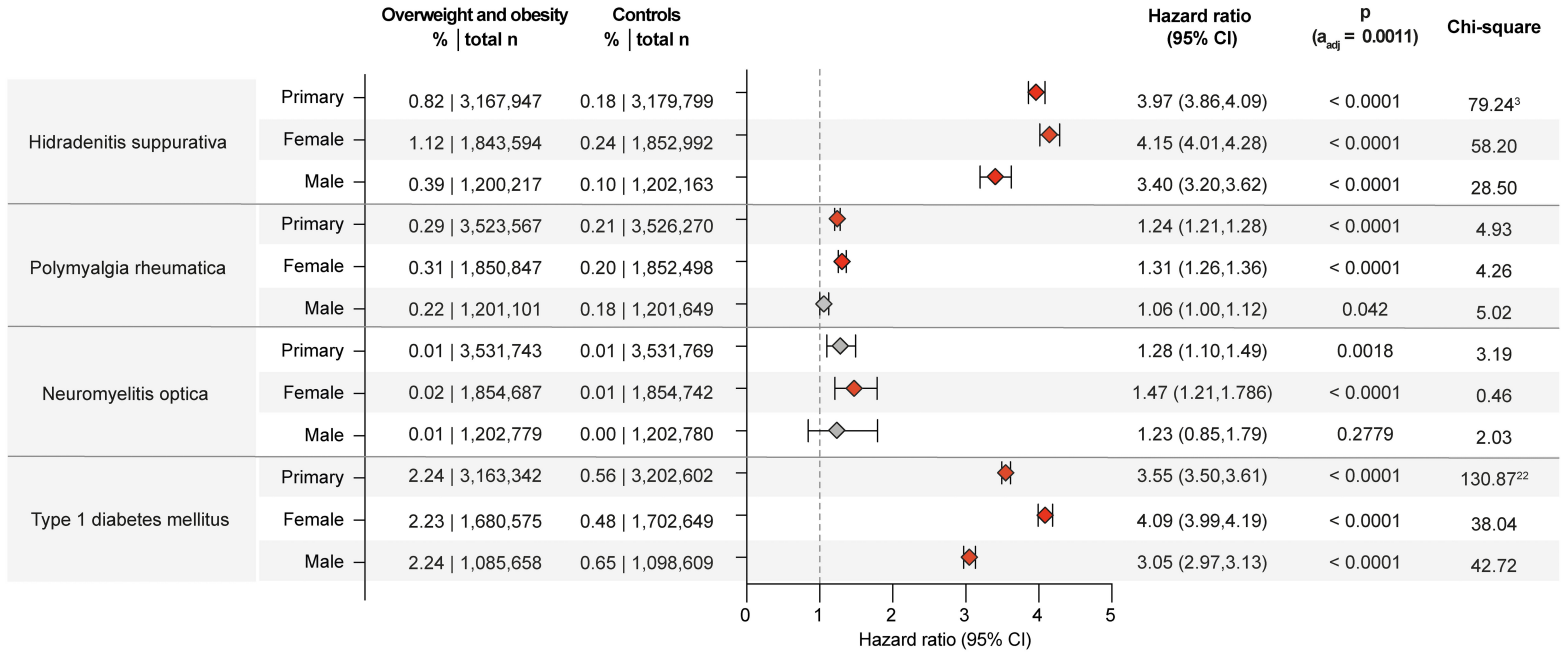
Chronic non-communicable inflammatory diseases with sex disparities in obesity and overweight-related risks. Risk of those chronic non-communicable inflammatory diseases that demonstrated sex disparities relating to the risk imposed by obesity and overweight. For comparisons with a highly violated proportionality assumption in the primary analysis, the odds ratio (OR) that also excludes outcomes prior to the index event, its’ 95%-confidence interval and p-value are provided in the footnotes: ^3^OR 4.558 (4.43,4.69) p<0.0001, ^22^OR 4.071 (4.005,4.139) p<0.0001. Please note that numbering of footnotes is based on **Supplementary Table 2**.

**Figure 4:**
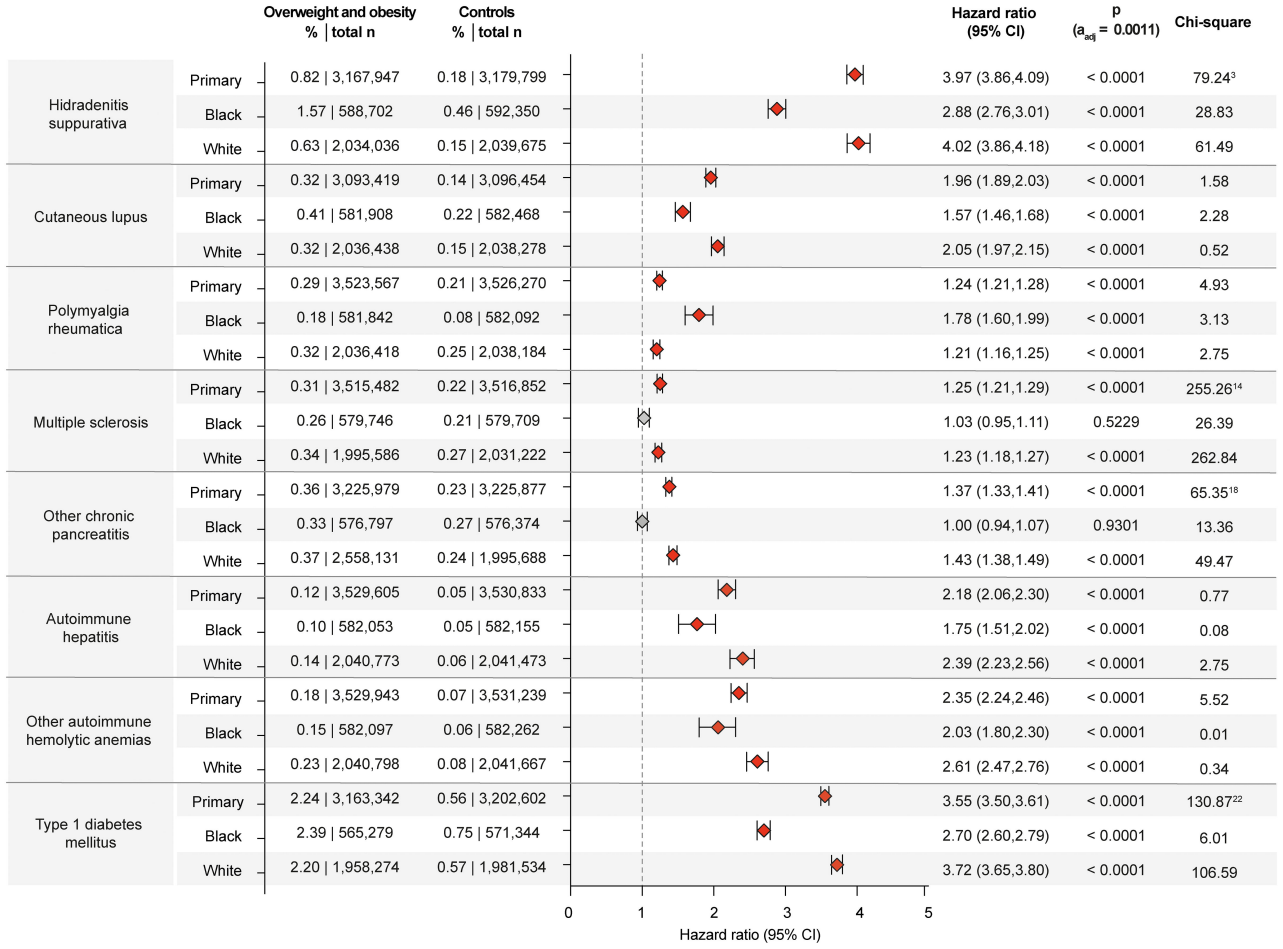
Chronic non-communicable inflammatory diseases with racial disparities in obesity and overweight-related risks. Risk of those chronic non-communicable inflammatory diseases that demonstrated racial disparities relating to the risk imposed by obesity and overweight. For comparisons with a highly violated proportionality assumption in the primary analysis, the odds ratio (OR) that also excludes outcomes prior to the index event, its’ 95%-confidence interval and p-value are provided in the footnotes: ^3^OR 4.558 (4.43,4.69) p<0.0001, ^14^OR 1.412 (1.371,1.454) p<0.0001, ^18^OR 1.871 (1.863,1.879) p<0.0001, ^22^OR 4.071 (4.005,4.139) p<0.0001. Please note that numbering of footnotes is based on **Supplementary Table 2**.

While obesity is a well-known risk factor for hidradenitis suppurativa (34), there are scant insights into sex- and racial disparities (35, 36). Herein, obesity is confirmed as risk factor for hidradenitis suppurativa across the primary and both sensitivity analyses (**Supplementary Table 2**). Relating to sex- and racial disparities, obesity and overweight imposed higher risks in females (HR 4.15, CI 4.01-4.28, p<0.0001) compared to males (HR 3.40, CI 3.20-3.62, p<0.0001) as well as in those of White ancestry (HR 4.02, CI 3.86-4.18, p<0.0001) compared to those of “Black or African American” ancestry (HR 2.88, CI 2.76-3.01, p<0.0001, **Figures 3**, **4**). A small-scale study indicated that obesity is not a risk factor for vitiligo (37). In contrast, another study found that metabolic syndrome is more prevalent in individuals with vitiligo compared to non-vitiligo controls (38). In the primary analysis, 0.33% of obese and overweight individuals were subsequently diagnosed with vitiligo, as opposed to 0.16% in the controls (HR 1.79, CI 1.73-1.85, p<0.0001). The results were consistent across all sensitivity, sex-, and race-stratified analyses, with no apparent sex or racial disparities. There is only one report demonstrating an increased co-occurrence of generalized pustular psoriasis with obesity (39). The data presented here documents an increased risk for pustular psoriasis in individuals with obesity (HR 1.54, CI 1.49-1.59, p<0.0001). The results remained consistent across all sensitivity analyses, as well as sex- and race-stratified analyses, showing no apparent sex or racial disparities. The incidence of prurigo nodularis PN was higher in the overweight and obese population (0.37%) compared to the non-obese population (0.19%), translating in a significant risk increment (HR 1.77, CI1.72-1.81, p<0.0001). Again, all sensitivity, sex- and race-stratified analysis showed almost identical results. Obesity also conferred increased risks for urticaria (HR 1.72, CI 1.71-1.74, p<0.0001) and cutaneous lupus (HR 1.96, CI 1.89-2.03, p<0.0001) across all analyses, conditions for which no previous data were available. Additionally, a racial disparity was noted in the risk of cutaneous lupus, with the risk being more pronounced in EHRs with White ancestry (HR 2.05, CI 1.97-2.15, p<0.0001) compared to those with “Black of African American” descent (HR 1.57, CI 1.46-1.68, p<0.0001, **Figure 4**).

#### Rheumatic CIDs

Inconsistent results have been reported regarding the impact of obesity on systemic lupus erythematosus (SLE) (21). In this study, the primary analysis included a total of 3,083,416 EHRs in each group. Among these, 12,070 cases of SLE were identified in obese and overweight individuals, resulting in a risk of 0.39%, as opposed to 0.21% in non-obese/overweight controls (HR 1.63, CI 1.58-1.68, p<0.0001). The results were consistent across all sensitivity analyses, and no sex or racial disparities were observed. While obesity is associated with disease severity in patients with ankylosing spondylitis (40), previous studies have documented that obesity does not confer an increased risk of developing the disease (41). In contrast, the data presented herein show an increased risk of ankylosing spondylitis among the obese population (HR 1.67, CI 1.60-1.74, p<0.0001). Similar results were obtained in all sensitivity and stratified analyses. Relating to sarcoidosis inconsistent results on the impact of obesity and disease risk have been published. A large-scale retrospective study using data from the US Veterans Health Administration database demonstrated that an increased BMI is not significantly associated with higher odds of developing sarcoidosis (42). In contrast, three previous studies have documented that individuals with obesity or weight gain are at a higher risk of developing sarcoidosis (43–45). In the data presented here, a total of 14,419 sarcoidosis patients were included, making this the largest investigation relating to the risk of overweight and obesity on sarcoidosis to date. Among the overweight and obese group, sarcoidosis was diagnosed in 9,582 cases (0.27%), while in the non-overweight/obese control group, 4,837 cases (0.14%) were diagnosed with sarcoidosis. The primary analysis for sarcoidosis showed that the risk associated with obesity was significantly elevated. The HR amounted to 1.75 (CI 1.69-1.82, p<0.000. This heightened risk remained consistent across all sensitivity analyses (**Supplementary Table 2**). Furthermore, as detailed in **Supplementary Table 2**, data from the US Collaborative Network documents a significant association between obesity and an increased risk of dermatomyositis. Among overweight and obese individuals 2,567 (0.073%) cases of dermatomyositis were documented, whilst this amounted to 0.04% in non-overweight/obese controls (HR 1.60, CI 1.50-1.71, p<0.0001). The results were consistent across all other analyses, with no significant differences related to sex or ancestry.

#### Respiratory CIDs

The impact of body weight on chronic obstructive pulmonary disease (COPD) risk, disease severity, and patient outcomes is complex (46). Studies focusing on the risk of BMI on COPD risk show divergent results. Some studies noted that an increase in waist circumference or abdominal adiposity were risk factors in COPD (47, 48). By contrast, the same study and others demonstrate that underweight is also associated with an increased COPD risk (48, 49). Others documented no impact of metabolic syndrome, which includes overweight and obesity, showed no risk differences for subsequent COPD development (50). In alignment with this inconclusive data, a mendelian randomization study concluded that obesity might also increase the susceptibility to COPD (51). In the primary analysis of this study documents COPD in 5.11% overweight/obese individuals, compared to 2.67% in the non-overweight/obese group. This represents a significant difference in risk between the two populations (HR 1.7, CI 1.686-1.714, p<0.0001, **Supplementary Table 2**).

#### Endocrine CIDs

Prospective studies have reported a twofold increased risk of type 1 diabetes in obese children and obese adults, especially in latent autoimmune diabetes (9, 52, 53). Another study, however, found no increased risk of overweight and obese individuals for type 1 diabetes (54). The data of this study supports the notion that overweight and obesity are risk factors for the development of type 1 diabetes mellitus, In detail, the primary analysis showed a significant increase in type 1 diabetes mellitus risk associated with obesity. Among the overweight and obese group, the risk was 2.24%, compared to 0.56% in the non-overweight/obese control group. This risk difference translates into a HR of 3.55 (CI 3.50-3.61, p<0.0001). This result was consistently observed in both sensitivity analyses. In the sex-stratified analysis for Type 1 diabetes mellitus, the risk associated with obesity was higher in females compared to males. For females, the risk was 2.45% (HR 3.85, CI 3.75-3.95, p<0.0001). In comparison, the risk for males was 1.95% (HR 3.14, CI 3.06-3.22, p<0.0001). Similar disparities were observed in the race-stratified analysis. Here the risk of developing type 1 diabetes mellitus following documentation of obesity is higher in Black or African American individuals (HR 4.55, CI 4.32-4.79, p<0.0001) compared to White individuals (HR 3.25, CI 3.18-3.33, p<0.0001, **Figure 4**, **Supplementary Table 2**). Likewise, in autoimmune thyroiditis inconsistent results on the impact of overweight and obesity have been reported (55). Herein, the primary analysis for autoimmune thyroiditis revealed a risk of 0.90% in the overweight and obese group, compared to 0.55% in the non-overweight/obese group. This difference results in a HR of 1.44 (CI 1.41-1.47, p<0.0001, **Supplementary Table 2**). No sex- or racial-disparities were noted regarding the risk for autoimmune thyroiditis.

#### Gastrointestinal CIDs

To date, overweight and obesity have not been established as recognized risk factors for autoimmune hepatitis. In this study, an increased risk of autoimmune hepatitis was documented in overweight individuals compared to non-overweight controls. Specifically, the risk of autoimmune hepatitis in the overweight and obese group was 0.122%, while in the non-overweight control group, the risk was 0.05%. This resulted in a statistically significant difference (HR 2.18, CI 2.06-2.30, p<0.0001). The results from the primary analysis persisted in both sensitivity analyses. The risk for autoimmune hepatitis imposed by overweight and obesity was more pronounced in White individuals (HR 2.39, CI 2.23-2.56, p<0.0001) compared to those of Black or African American descent (HR 1.75, CI1.51-2.02, p<0.0001, **Figure 4**). No sex-specific impact on this risk was noted (**Supplementary Table 2**).

#### Neurological CIDs

Overweight and obesity are well-recognized risk factors for multiple sclerosis (56). Findings of this study are in alignment with these previous results. In addition, the increased risk for multiple sclerosis imposed by overweight and obesity was observed in White individuals but not in Black or African American individuals. In White individuals, the risk of MS was 0.34% (HR 1.23, CI 1.18-1.27, p<0.0001). In contrast, in Black or African American individuals, overweight and obesity did not change the risk for a subsequent multiple sclerosis diagnosis (HR 1.02, CI 0.95-1.106, p= 0.5229 **Supplementary Table 2**). No significant sex differences were observed in multiple sclerosis risk imposed by overweight and obesity.

#### Haematological CIDs

Whilst overweight and obesity potentially increased the risk for thromboembolic disease in patients with antiphospholipid syndrome (57), its impact on the risk for disease manifestation remained uncertain. Here, overweight and obesity were associated with a higher risk of developing antiphospholipid syndrome compared to non-overweight controls (HR 2.15, CI 2.07-2.23, p<0.0001). Results were consistent across all sensitivity, age-, and race-stratified analyses, with no apparent risk differences observed between sexes or racial groups (**Supplementary Table 2**). Regarding autoimmune hemolytic anemia and pernicious anemia, the impact on their risk imposed by overweight and obesity had not been know. Here, an increased risk for both diseases in overweigh and obese compared to non-overweight/obese controls is documented. In addition, the risk imposed by overweight and obesity was more pronounced in White as opposed to Black or African American individuals (**Figure 4**). Detailed results are shown in **Supplementary Table 2**.

### Only primary and one sensitivity analysis identify obesity as a risk factor for 12 of 46 investigated CIDs

In the primary analysis, the results demonstrated an increased risk for developing several additional CIDs in the overweight/obese population. Specifically, the analysis showed a significant increase in the risk for pyoderma gangrenosum, atopic dermatitis, lichen sclerosus, pemphigus diseases, pemphigoid diseases, dermatitis herpetiformis, polyarteritis nodosa, polymyalgia rheumatica, systemic vasculitis, immune thrombocytopenic purpura, myasthenia gravis, and primary biliary cirrhosis. These results were also seen in sensitivity analysis S2. However, in the sensitivity analysis S1, where BMI rather than a diagnosis of overweight and obesity was used, this increased risk was not validated (**Supplementary Table 2**). Lack of validation in one of the sensitivity analyses challenges the findings of the primary analysis. Thus, obesity should not be considered a risk factor for these CIDs. Of note, among these CIDs, where overweight may be a risk factor, in the case of polymyalgia rheumatica, the increased risk was more pronounced in females and individuals of Black or African American ancestry. Specifically, the risk for women is HR 1.308 (CI 1.255-1.363, p<0.0001), whilst this amounted to a HR of 1.06 (CI 1.002-1.122, p=0.0420) in men. In Black or African American individuals, the risk amounted to a HR 1.78 (CI 1.60-2.00, p<0.0001), compared to White individuals, demonstrating a HR of 1.21 (CI 1.16-1.25, p<0.0001).

### Obesity is not a risk factor for 10 of 46 investigated CIDs

No impact of obesity on the risk of developing alopecia areata, localized scleroderma (morphea), lichen planus, systemic sclerosis, Sjögren syndrome, neuromyelitis optica, celiac disease, Crohn’s disease, ulcerative colitis, or other chronic pancreatitis was noted herein. Specifically, because no significant risk of obesity was noted in the primary analysis, lack of validation in both sensitivity analyses, or opposing results in the sensitivity analyses (**Supplementary Table 2**). In the sensitivity analysis S1, where BMI rather than the diagnosis of overweight and obesity was used as the determinant, the associations observed in the primary analysis were not confirmed. Amongst these CIDs, overweight and obesity conferred an increased risk for neuromyelitis optica restricted to in females, with a HR of 1.47 (CI 1.21-1.79, p<0.0001). By contrast, the risk in males was not significant, with a HR amounting to 1.23 (CI 0.85-1.79, p=0.2779).

## Discussion

This study thoroughly documents the impact of obesity on the risk for CIDs. Overweight and obesity increased the risk for the subsequent clinical manifestation of 52% of the CIDs investigated. Additionally, for 26% of the diseases, obesity emerged as a potential risk factor for future CID manifestation. For 22% of CIDs, no consistent association with overweight or obesity was identified. The large sample size enabled an in-depth examination of sex- and race-related disparities in the potential risk posed by overweight and obesity on CID manifestation. In sex-stratified analysis, overweight and obesity conferred a more pronounced risk for four CIDs in female individuals. In race-stratified analysis, overweight and obesity were associated with a higher risk for seven CIDs in White individuals and one CID in Black or African American individuals.

From the 46 investigated CIDs, four discordant results were observed when analyzing the obesity-imposed risk for CID for women and men separately. The heightened immune response in women may contribute to a predisposition for the loss of self-tolerance and the subsequent development of autoimmune diseases, as evidenced by the significant female predominance among patients with CIDs (58). Overweight and obesity may potentially exacerbate this risk imposed by female sex, further increasing the likelihood of developing CIDs. This highlights the importance of tailored weight management strategies and monitoring especially in women at risk for CIDs to mitigate the additional risk of obesity on CID manifestation. These sex differences relating to CID risk is potentially related to the different immune profile (59) and/or the different susceptibility to CIDs (60) of women and men. Similarly to the sex-specific impact of overweight and obesity on CID risk, racial disparities on this topic have been rarely addressed. The large sample size of this study allowed a race-stratified analysis, to investigate potential racial disparities on the overweight/obesity-imposed risk for CID development. In detail, overweight and obesity were linked to a higher risk for seven CIDs in White individuals and one CID in Black or African American individuals. Again, like the herein reported sex differences, insights into racial disparities of the overweight/obesity-imposed risk on CID risk, allow tailored preventive measures in those populations where obesity confers a higher risk for a subsequent CID diagnosis. Potential explanations for the observed racial disparities are manifold, including genetics, but at least equally important, socioeconomic factors, healthcare access and treatment disparities (61–63).

The molecular basis for the here observed, obesity-imposed increased risk for CIDs remains to be fully elucidated. We assume that the interaction between obesity and inflammation is complex and most likely differs between different diseases. For example, in SLE, Vitamin D and adipokines potentially from a pathogenic link between obesity and disease manifestation (3, 55). Furthermore, the intestinal microbiome has been recognized as a potential link between obesity and SLE: In mice fed different diets, those on a Western Diet had an earlier onset and an overall higher incidence of lupus nephritis compared to mice on control chow. By contrast, mice on caloric restriction were completely protected from lupus nephritis. Of note, at a time where all mice were phenotypically healthy, the diet-induced changes of the intestinal microbiome allowed to predict future disease manifestation (64). Similar observations were made in the same strain of mice investigating the risk of autoimmune pancreatitis (65). Obesity, gastrointestinal dysbiosis and dietary habits have jointly been linked to contribute to low-grade systemic chronic inflammation. Consequences of this sustained, low-grade inflammatory state are a number of complex diseases, including CIDs (66).

Research on weight loss interventions supports the idea that obesity may not only be linked to CIDs but could also play a causative role. A recent study by Dr Ma and colleagues (67) evaluated the effects of bariatric surgery in patients with rheumatoid arthritis and SLE. The findings revealed a significant reduction in the need for immunosuppressant medications over a mean follow-up period of 19 months. Similar outcomes have been reported in inflammatory bowel disease, where patients undergoing bariatric surgery experienced fewer IBD-related complications compared to matched controls (68). Notably, the strongest evidence for the impact of weight loss on disease severity is seen in psoriasis (69). Given the approval of glucagon-like peptide-1 (GLP-1) analogues for weight loss (70), similar effects can be expected from these compounds. A manuscript currently under submission from our laboratory supports this assumption, demonstrating the long-term beneficial effects of GLP-1 analogue treatment in patients with psoriasis undergoing systemic therapy (*Olbrich* et al*, submitted*).

Our study has several limitations that need to be acknowledged: First, the definition of diagnoses and outcomes is solely based on ICD-10 codes. Data quality relies on the quality of coding and does not rule out bias imposed by non- or miscoding. To partially address this limitation, data from HCOs undergoes stringent harmonization, standardization and checks for plausibility before uploading on the federated database. However, there is no firm validation of the diagnoses. Second, the retrospective data collection is a methodological limitation and potential confounder. Third, we have not analyzed a “dose-dependency” effect, e.g., the magnitude of obesity was not considered. Along the line, the severity of the CIDs can, so far, not be determined in TriNetX. Thus, extreme phenotypes with a significant impact on CID risk could potentially confound the results. Fourth, while large sample sizes are in general preferable, especially when investigating rare diseases, this may lead to an overestimation of effects because with these large sample sizes, relatively small effects are more likely to be considered significant. Thus, for some of the outcomes, especially those where sex-specific differences were noted or where the effect size was relatively small, metanalysis would be ideal to better investigate the impact of obesity on CID risk. However, due to data protection, the smallest possible cohorts in TriNetX are grouped per continents. Fifth, we used PSM to reduce confounding bias. For this, covariates that potentially influence the CID risk were selected. This, however, does not control for unmeasured or unknown confounders. PSM used a caliper distance of 0.1 that balances close matching whilst retaining a sufficient sample size. Change of the caliper distance is not possible in the automated analytical pipeline provided by TriNetX. Despite these limitations inherent to PSM, it is potentially advantageous over conventional covariate adjustment methods (31). Furthermore, potential bias stemming from unmeasured or unaccounted bias cannot be fully accounted for. For example, some potentially important possible confounders are not, or only rarely, documented in the TriNetX database, i.e., socioeconomic status, physical activity, dietary habits. To partially address this, several sensitivity analyses were employed. Lack of validation of several individual CIDs in the sensitivity analysis S1, where obesity was defined by BMI rather than the corresponding ICD10-code underscores the necessity of sensitivity analyses in retrospective cohort studies. We here interpreted lack of validation of the results from the primary analysis in any sensitivity analysis as no risk of obesity. Yet, this does not exclude more lenient interpretations of the data. In line with this notion, it is important to mention that the data presented herein does not allow to conclude on causality. To infer causality data analysis of prospectively collected registries is required. Alternatively, the impact of weight loss could on CID disease severity could be investigated in controlled clinical trials. Promising results have recently been reported in patients with inflammatory bowel disease where use of glucagon-like peptide-1 analogs (GLP-1RA) has been demonstrated to be associated with improved disease outcomes (71). In line with this data, we recently observed that GLP-1RA treatment in patients with psoriasis was safe and associated with reduced risks of cardiovascular and psychiatric comorbidities, as well as lower mortality (Olbrich et al., unpublished).

Despite these limitations, we believe our data to be clinically relevant because we validated previously well-established findings, such as the obesity-imposed risk increase for type 2 diabetes, psoriasis, asthma, and hidradenitis suppurativa (7–10, 32). Given the here-observed risk for CID development in obese individuals, measures for prevention or treatment of obesity hold the potential to prevent the clinical manifestation not only of metabolic and cardiovascular disease, but also a large number of CIDs. Given the high and largely unmet medical need imposed by these diseases on affected patients and the health-care system (6, 72), these preventive measures could have a significant impact.

## Data Availability

The original contributions presented in the study are included in the article/**Supplementary Material**. Further inquiries can be directed to the corresponding author/s.
