# Supplementary material for: Large-scale analysis highlights obesity as a risk factor for chronic, non-communicable inflammatory diseases
Source: Front Endocrinol (Lausanne). 2025 Feb 3;16:1516433. doi: 10.3389/fendo.2025.1516433 (PMC11830592; doi:10.3389/fendo.2025.1516433)
Supplement: Supplementary file 1 [file Table1.docx]

| **Medical field** | **Endpoint** | **Diagnoses (if composite endpoint)** | **ICD10-codes** |
| --- | --- | --- | --- |
| **Dermatology** | Pyoderma gangrenosum | - | ICD10CM:L88 |
|  | Hidradenitis suppurativa | - | ICD10CM:L73.2 |
|  | Alopecia areata | - | ICD10CM:L63 |
|  | Vitiligo | - | ICD10CM:L80 |
|  | Psoriasis vulgaris | - | ICD10CM:L40.0 |
|  | Pustular psoriasis | - Generalized pustular psoriasis - Pustulosis palmaris et plantaris - Acrodermatitis continua | ICD10CM:L40.1  ICD10CM:L40.3  ICD10CM:L40.2 |
|  | Psoriatic arthritis | - | ICD10CM:L40.5 |
|  | Atopic dermatitis | - | ICD10CM:L20 |
|  | Prurigo nodularis | - | ICD10CM:L28.1 |
|  | Lichen planus | - | ICD10CM:L43 |
|  | Localized scleroderma [morphea] | - | ICD10CM:L94.0 |
|  | Urticaria | - | ICD10CM:L50 |
|  | Lichen sclerosus | - | ICD10CM:L90.0 |
|  | Cutaneous lupus | - | ICD10CM:L93 |
|  | Pemphigus diseses | - Pemphigus vulgaris - Pemphigus vegetans - Pemphigus foliaceous - Pemphigus erythematosus | ICD10CM:L10.0  ICD10CM:L10.1  ICD10CM:L10.2  ICD10CM:L10.4 |
|  | Pemphigoid diseases | - Bullous pemphigoid - Cicatricial pemphigoid - Acquired epidermolysis bullosa - Other pemphigoid - Pemphigoid, unspecified | ICD10CM:L12.0  ICD10CM:L12.1  ICD10CM:L12.3  ICD10CM:L12.8  ICD10CM:L12.9 |
|  | Dermatitis herpetiformis | - | ICD10CM:L13.0 |
| **Systemic rheumatic diseases** | Systemic lupus erythematosus (SLE) | - | ICD10CM:M32 |
|  | Systemic sclerosis | - | ICD10CM:M34 |
|  | Rheumatoid arthritis | Rheumatoid arthritis with rheumatoid factor  Other rheumatoid arthritis | ICD10CM:M05  ICD10CM:M06 |
|  | Sjögren syndrome | - | ICD10CM:M35.0 |
|  | Ankylosing spondylitis | - | ICD10CM:M45 |
|  | Polyarteritis nodosa | - | ICD10CM:M30.0 |
|  | Polymyalgia rheumatica | - | ICD10CM:M35.3 |
|  | Dermatomyositis | - | ICD10CM:M33 |
|  | Sarcoidosis | - | ICD10CM:D86 |
|  | Systemic vasculits | Granulomatosis with polyangiitis (GPA)  Eosinophilic GPA (EGPA)  Microscopic polyangiitis | ICD10CM:M31.3  ICD10CM:M30.1  ICD10CM:M31.7 |
| **Neurology** | Myasthenia gravis | - | ICD10CM:G70.0 |
|  | Multiple sclerosis | - | ICD10CM:G35 |
|  | Neuromyelitis optica [Devic] | - | ICD10CM:G36.0 |
| **Gastoenerology** | Celiac disease | - | ICD10CM:K90.0 |
|  | Crohn's disease [regional enteritis] | - | ICD10CM:K50 |
|  | Ulcerative colitis | - | ICD10CM:K51 |
|  | Primary biliary cirrhosis | - | ICD10CM:K74.3 |
|  | Other chronic pancreatitis | - | ICD10CM:K86.1 |
|  | Autoimmune hepatitis | - | ICD10CM:K75.4 |
| **Haematology** | Vitamin B12 deficiency anemia due to intrinsic factor deficiency | - | ICD10CM:D51.0 |
|  | Other autoimmune hemolytic anemias | - | ICD10CM:D59.1 |
|  | Immune thrombocytopenic purpura | - | ICD10CM:D69.3 |
|  | Antiphospholipid syndrome | - | ICD10CM:D68.61 |
| **Endocrinology** | Autoimmune thyroiditis | - | ICD10CM:E06.3 |
|  | Type 1 diabetes mellitus | - | ICD10CM:E10 |
| **Ear-nose and throat** | Chronic sinusitis | - | ICD10CM:J32 |
|  | Vasomotor and allergic rhinitis | - | ICD10CM:J30 |
| **Pulmology** | Asthma | - | ICD10CM:J45 |
|  | Other chronic obstructive pulmonary disease | - | ICD10CM:J44 |

**Supplement Table 1.** Definition of outcomes.

| **Outcome** | **Analysis** | **Overweight and obesity**  **(Cases)** | | | **Controls** | | | **Results** | | | | | |
| --- | --- | --- | --- | --- | --- | --- | --- | --- | --- | --- | --- | --- | --- |
|  |  | **N of eligible participants** | **N of Out-comes** | **Risk, %** | **N of eligible participants** | **N of Out-comes** | **Risk, %** | **Risk difference, %** | **(95% confidence interval)** | **Hazard ratio** | **(95% confidence interval)** | **P value**  **α_adj_.=0.0011** | **Chi-square** |
| **T2DM** | Primary | 2,558,131 | 371,053 | 14.505 | 2,955,679 | 142,959 | 4.837 | 9.668 | (9.618,9.718) | 2.845 | (2.827,2.862) | < 0.0001 | 290.017^1^ |
|  | S1 | 910,155 | 151,779 | 16.676 | 976,585 | 68,432 | 7.007 | 9.669 | (9.577,9.761) | 2.432 | (2.41,2.454) | < 0.0001 | 92.004 |
|  | S2 | 2,496,948 | 226,887 | 9.087 | 2,921,641 | 81,947 | 2.805 | 6.282 | (6.241,6.322) | 2.925 | (2.902,2.948) | < 0.0001 | 180.978 |
|  | Female | 1,390,812 | 207,739 | 14.937 | 1,592,767 | 69,971 | 4.393 | 10.543 | (10.476,10.611) | 3.185 | (3.158,3.212) | < 0.0001 | 56.056 |
|  | Male | 847,444 | 129,328 | 15.261 | 997,094 | 56,458 | 5.662 | 9.599 | (9.51,9.688) | 2.555 | (2.53,2.581) | < 0.0001 | 134.009 |
|  | Black | 451,950 | 70,830 | 15.672 | 513,589 | 30,464 | 5.932 | 9.74 | (9.616,9.865) | 2.368 | (2.336,2.4) | < 0.0001 | 22.963 |
|  | White | 1,580,882 | 237,184 | 15.003 | 1,833,855 | 93,484 | 5.098 | 9.906 | (9.841,9.97) | 3.032 | (3.009,3.055) | < 0.0001 | 138.335 |
| **Any CID** | Primary | 2,047,553 | 583,068 | 28.476 | 2,498,251 | 438,336 | 17.546 | 10.931 | (10.853,11.008) | 1.515 | (1.509,1.521) | < 0.0001 | 198.85^2^ |
|  | S1 | 748,591 | 253,564 | 33.872 | 793,676 | 217,696 | 27.429 | 6.443 | (6.298,6.589) | 1.221 | (1.214,1.228) | < 0.0001 | 9.819 |
|  | S2 | 1,967,309 | 410,114 | 20.846 | 2,432,455 | 299,822 | 12.326 | 8.521 | (8.45,8.591) | 1.512 | (1.505,1.519) | < 0.0001 | 75.384 |
|  | Female | 1,044,647 | 332,763 | 31.854 | 1,302,123 | 245,218 | 18.832 | 13.022 | (12.91,13.134) | 1.583 | (1.575,1.591) | < 0.0001 | 96.62 |
|  | Male | 747,134 | 184,030 | 24.631 | 885,598 | 137,025 | 15.473 | 9.159 | (9.035,9.282) | 1.464 | (1.454,1.474) | < 0.0001 | 54.72 |
|  | Black | 371,045 | 108,262 | 29.178 | 447,470 | 77,020 | 17.212 | 11.965 | (11.782,12.149) | 1.565 | (1.551,1.58) | < 0.0001 | 55.13 |
|  | White | 1,246,212 | 359,906 | 28.88 | 1,512,313 | 294,086 | 19.446 | 9.434 | (9.332,9.535) | 1.497 | (1.49,1.505) | < 0.0001 | 161.428 |
| **Pyoderma gangrenosum** | Primary | 3,184,225 | 1,105 | 0.035 | 3,184,524 | 426 | 0.013 | 0.021 | (0.019,0.024) | 2.267 | (2.028,2.536) | < 0.0001 | 1.343 |
|  | S1 | 1,073,644 | 423 | 0.039 | 1,073,699 | 369 | 0.034 | 0.005 | (0,0.01) | 1.083 | (0.942,1.246) | 0.2616 | 1.212 |
|  | S2 | 3,184,117 | 658 | 0.021 | 3,184,493 | 275 | 0.009 | 0.012 | (0.01,0.014) | 2.056 | (1.786,2.367) | < 0.0001 | 1.092 |
|  | Female | 1,856,379 | 709 | 0.038 | 1,856,566 | 296 | 0.016 | 0.022 | (0.019,0.026) | 2.09 | (1.825,2.393) | < 0.0001 | 7.201 |
|  | Male | 1,203,133 | 357 | 0.03 | 1,203,228 | 145 | 0.012 | 0.018 | (0.014,0.021) | 2.194 | (1.809,2.661) | < 0.0001 | 0.535 |
|  | Black* | 594,615 | 209 | 0.035 | 594,641 | 88 | 0.015 | 0.02 | (0.015,0.026) | 1.981 | (1.544,2.542) | < 0.0001 | 1.091 |
|  | White | 2,041,818 | 743 | 0.036 | 2,042,018 | 318 | 0.016 | 0.021 | (0.018,0.024) | 2.208 | (1.936,2.518) | < 0.0001 | 6.195 |
| **Hidradenitis suppurativa** | Primary | 3,167,947 | 26,109 | 0.824 | 3,179,799 | 5,787 | 0.182 | 0.642 | (0.631,0.653) | 3.971 | (3.86,4.086) | < 0.0001 | 79.243^3^ |
|  | S1 | 1,069,398 | 10,125 | 0.947 | 1,072,560 | 1,987 | 0.185 | 0.762 | (0.741,0.782) | 4.837 | (4.61,5.075) | < 0.0001 | 25.091 |
|  | S2 | 3,166,098 | 16,520 | 0.522 | 3,179,216 | 3,742 | 0.118 | 0.404 | (0.395,0.413) | 3.821 | (3.687,3.959) | < 0.0001 | 14.823 |
|  | Female | 1,843,594 | 20,620 | 1.118 | 1,852,992 | 4,388 | 0.237 | 0.882 | (0.865,0.898) | 4.146 | (4.013,4.284) | < 0.0001 | 58.2 |
|  | Male | 1,200,217 | 4,663 | 0.389 | 1,202,163 | 1,223 | 0.102 | 0.287 | (0.274,0.299) | 3.403 | (3.195,3.624) | < 0.0001 | 28.497 |
|  | Black* | 588,702 | 9,235 | 1.569 | 592,350 | 2,713 | 0.458 | 1.111 | (1.075%1.147) | 2.881 | (2.76,3.007) | < 0.0001 | 28.834 |
|  | White | 2,034,036 | 12,842 | 0.631 | 2,039,675 | 3,030 | 0.149 | 0.483 | (0.471,0.495) | 4.019 | (3.863,4.182) | < 0.0001 | 61.492 |
| **Alopecia areata** | Primary | 3,180,021 | 5,778 | 0.182 | 3,181,755 | 3,762 | 0.118 | 0.063 | (0.057,0.069) | 1.341 | (1.287,1.398) | < 0.0001 | 2.509 |
|  | S1 | 1,072,522 | 2,069 | 0.193 | 1,072,544 | 2,194 | 0.205 | -0.012 | (-0.024,0) | 0.89 | (0.838,0.945) | 0.0001 | 0.055 |
|  | S2 | 3,179,638 | 3,687 | 0.116 | 3,181,452 | 2,381 | 0.075 | 0.041 | (0.036,0.046) | 1.332 | (1.265,1.403) | < 0.0001 | 0.152 |
|  | Female | 1,853,470 | 4,162 | 0.225 | 1,854,745 | 2,651 | 0.143 | 0.082 | (0.073,0.09) | 1.369 | (1.304,1.438) | < 0.0001 | 13.879 |
|  | Male | 1,202,013 | 1,368 | 0.114 | 1,202,466 | 991 | 0.082 | 0.031 | (0.023,0.039) | 1.226 | (1.129,1.33) | < 0.0001 | 0.124 |
|  | Black* | 593,642 | 1,449 | 0.244 | 594,058 | 832 | 0.14 | 0.104 | (0.088,0.12) | 1.456 | (1.336,1.585) | < 0.0001 | 4.43 |
|  | White | 2,039,643 | 3,052 | 0.15 | 2,040,498 | 2,254 | 0.11 | 0.039 | (0.032,0.046) | 1.277 | (1.209,1.348) | < 0.0001 | 1.144 |
| **Vitiligo** | Primary | 3,176,648 | 10,409 | 0.328 | 3,180,170 | 5,094 | 0.16 | 0.167 | (0.16,0.175) | 1.79 | (1.731,1.851) | < 0.0001 | 5.037 |
|  | S1 | 1,071,567 | 4,670 | 0.436 | 1,071,527 | 3,739 | 0.349 | 0.087 | (0.07,0.104) | 1.184 | (1.135,1.236) | < 0.0001 | 32.102 |
|  | S2 | 3,175,642 | 6,438 | 0.203 | 3,179,687 | 3,240 | 0.102 | 0.101 | (0.095,0.107) | 1.713 | (1.642,1.787) | < 0.0001 | 4.841 |
|  | Female | 1,852,039 | 6,623 | 0.358 | 1,854,025 | 3,049 | 0.164 | 0.193 | (0.183,0.204) | 1.902 | (1.823,1.986) | < 0.0001 | 5.711 |
|  | Male | 1,200,114 | 3,619 | 0.302 | 1,201,634 | 1,830 | 0.152 | 0.149 | (0.137,0.161) | 1.764 | (1.668,1.867) | < 0.0001 | 0.098 |
|  | Black* | 593,385 | 2,123 | 0.358 | 593,835 | 984 | 0.166 | 0.192 | (0.174,0.21) | 1.799 | (1.668,1.941) | < 0.0001 | 0.0012 |
|  | White | 2,036,821 | 6,979 | 0.343 | 2,039,345 | 3,298 | 0.162 | 0.181 | (0.171,0.191) | 1.999 | (1.918,2.084) | < 0.0001 | 0.623 |
| **Psoriasis vulgaris** | Primary | 3,163,729 | 15,924 | 0.503 | 3,172,714 | 8,410 | 0.265 | 0.238 | (0.229,0.248) | 1.659 | (1.616,1.703) | < 0.0001 | 70.305^4^ |
|  | S1 | 1,071,567 | 1,067,978 | 0.671 | 1,069,247 | 4,689 | 0.439 | 0.233 | (0.213,0.253) | 1.448 | (1.395,1.502) | < 0.0001 | 0.534 |
|  | S2 | 3,162,446 | 10,776 | 0.341 | 3,171,823 | 5,770 | 0.182 | 0.159 | (0.151,0.167) | 1.614 | (1.563,1.666) | < 0.0001 | 4.778 |
|  | Female | 1,846,443 | 8,490 | 0.46 | 1,850,870 | 4,358 | 0.235 | 0.224 | (0.212,0.236) | 1.709 | (1.647,1.772) | < 0.0001 | 29.271 |
|  | Male | 1,195,348 | 5,017 | 0.42 | 1,198,594 | 2,958 | 0.247 | 0.173 | (0.158,0.188) | 1.51 | (1.443,1.581) | < 0.0001 | 35.418 |
|  | Black* | 593,520 | 1,187 | 0.2 | 594,026 | 555 | 0.093 | 0.107 | (0.093,0.12) | 1.798 | (1.625,1.989) | < 0.0001 | 35.332 |
|  | White | 2,027,490 | 10,574 | 0.522 | 2,033,788 | 5,702 | 0.28 | 0.241 | (0.229,0.253) | 1.75 | (1.695,1.808) | < 0.0001 | 48.428 |
| **Pustular psoriasis** | Primary | 3,082,740 | 10,723 | 0.348 | 3,090,912 | 6,099 | 0.197 | 0.151 | (0.142,0.159) | 1.541 | (1.493,1.59) | < 0.0001 | 37.571^5^ |
|  | S1 | 1,070,520 | 5,417 | 0.506 | 1,071,603 | 3,908 | 0.365 | 0.141 | (0.124,0.159) | 1.312 | (1.259,1.367) | < 0.0001 | 0.011 |
|  | S2 | 3,206,689 | 7,532 | 0.235 | 3,215,116 | 4,372 | 0.136 | 0.099 | (0.092,0.106) | 1.497 | (1.442,1.554) | < 0.0001 | 0.319 |
|  | Female | 1,846,488 | 5,193 | 0.32 | 1,850,415 | 3,409 | 0.184 | 0.136 | (0.126,0.146) | 1.522 | (1.459,1.587) | < 0.0001 | 15.068 |
|  | Male | 1,195,901 | 2,961 | 0.148 | 1,198,751 | 1,963 | 0.164 | 0.084 | (0.072,0.095) | 1.341 | (1.266,1.42) | < 0.0001 | 22.371 |
|  | Black | 582,897 | 812 | 0.139 | 583,400 | 470 | 0.081 | 0.059 | (0.047,0.071) | 1.442 | (1.287,1.616) | < 0.0001 | 7.317 |
|  | White | 2,028,288 | 6,875 | 0.339 | 2,033,677 | 4,176 | 0.205 | 0.134 | (0.123,0.144) | 1.548 | (1.489,1.608) | < 0.0001 | 29.696 |
| **Psoriatic arthritis** | Primary | 3,173,607 | 10,376 | 0.327 | 3,178,518 | 4,720 | 0.148 | 0.178 | (0.171,0.186) | 1.925 | (1.86,1.992) | < 0.0001 | 8.807 |
|  | S1 | 1,071,322 | 3,836 | 0.358 | 1,072,199 | 2,001 | 0.187 | 0.171 | (0.157,0.185) | 1.813 | 1.718,1.914) | < 0.0001 | 4.313 |
|  | S2 | 3,172,797 | 6,630 | 0.209 | 3,178,070 | 2,974 | 0.094 | 0.115 | (0.109,0.121) | 1.922 | (1.84,2.007) | < 0.0001 | 6.296 |
|  | Female | 1,850,226 | 6,655 | 0.36 | 1,853,361 | 2,938 | 0.159 | 0.201 | (0.191,0.212) | 1.983 | (1.899,2.071) | < 0.0001 | 0.137 |
|  | Male | 1,199,240 | 3,193 | 0.266 | 1,200,859 | 1,761 | 0.147 | 0.12 | (0.108,0.131) | 1.615 | (1.524,1.712) | < 0.0001 | 3.954 |
|  | Black* | 594,226 | 741 | 0.125 | 594,418 | 283 | 0.048 | 0.077 | (0.067,0.088) | 2.18 | (1.901,2.501) | < 0.0001 | 3.2 |
|  | White | 2,033,157 | 8,107 | 0.399 | 2,037,156 | 3,805 | 0.187 | 0.212 | (0.201,0.222) | 2.016 | (1.94,2.095) | < 0.0001 | 3.75 |
| **Atopic dermatitis** | Primary | 3,142,757 | 45,812 | 1.458 | 3,164,781 | 27,447 | 0.867 | 0.59 | (0.574,0.607) | 1.471 | (1.449,1.493) | < 0.0001 | 73.015^6^ |
|  | S1 | 1,061,218 | 15,431 | 1.454 | 1,062,418 | 14,063 | 1.324 | 0.13 | (0.099,0.162) | 1.036 | 1.012,1.06) | 0.0025 | 4.994 |
|  | S2 | 3,139,873 | 30,670 | 0.977 | 3,162,780 | 18,580 | 0.587 | 0.389 | (0.376,0.403) | 1.432 | (1.406,1.458) | < 0.0001 | 13.901 |
|  | Female | 1,830,618 | 29,205 | 1.595 | 1,844,242 | 16,973 | 0.92 | 0.675 | (0.652,0.698) | 1.518 | (1.49,1.547) | < 0.0001 | 64.137 |
|  | Male | 1,189,320 | 13,542 | 1.139 | 1,196,652 | 8,492 | 0.71 | 0.429 | (0.405,0.453) | 1.429 | (1.391,1.468) | < 0.0001 | 7.739 |
|  | Black* | 582,003 | 12,199 | 2.096 | 588,946 | 6,977 | 1.185 | 0.911 | (0.865,0.957) | 1.491 | (1.448,1.535) | < 0.0001 | 53.076 |
|  | White | 2,021,738 | 23,365 | 1.156 | 2,031,645 | 15,034 | 0.74 | 0.416 | (0.397,0.435) | 1.475 | (1.445,1.505) | < 0.0001 | 23.055 |
| **Prurigo nodularis** | Primary | 3,177,073 | 11,850 | 0.373 | 3,180,992 | 5,878 | 0.185 | 0.188 | (0.18,0.196) | 1.765 | (1.711,1.821) | < 0.0001 | 37.064^7^ |
|  | S1 | 1,071,584 | 4,471 | 0.417 | 1,071,891 | 3,421 | 0.319 | 0.098 | (0.082,0.114) | 1.236 | (1.182,1.293) | < 0.0001 | 23.736 |
|  | S2 | 3,176,390 | 7,296 | 0.23 | 3,180,539 | 3,703 | 0.116 | 0.113 | (0.107,0.12) | 1.696 | (1.63,1.765) | < 0.0001 | 2.666 |
|  | Female | 1,852,303 | 6,827 | 0.369 | 1,854,496 | 3,285 | 0.177 | 0.191 | (0.181,0.202) | 1.817 | (1.742,1.894) | < 0.0001 | 43.778 |
|  | Male | 1,200,551 | 4,235 | 0.353 | 1,201,966 | 2,097 | 0.174 | 0.178 | (0.165,0.191) | 1.806 | (1.714,1.903) | < 0.0001 | 14.635 |
|  | Black* | 593,278 | 2,291 | 0.386 | 593,883 | 1,088 | 0.183 | 0.203 | (0.184,0.222) | 1.758 | (1.635,1.889) | < 0.0001 | 40.319 |
|  | White | 2,037,416 | 7,452 | 0.366 | 2,039,904 | 3,792 | 0.186 | 0.18 | (0.17,0.19) | 1.863 | (1.792,1.938) | < 0.0001 | 20.124 |
| **Lichen planus** | Primary | 3,179,371 | 7,611 | 0.239 | 3,181,337 | 5,577 | 0.175 | 0.064 | (0.057,0.071) | 1.192 | (1.151,1.234) | < 0.0001 | 8.313 |
|  | S1 | 1,072,583 | 2,549 | 0.238 | 1,072,541 | 2,686 | 0.25 | -0.013 | (-0.026,0) | 0.896 | (0.849,0.946) | < 0.0001 | 5.694 |
|  | S2 | 3,178,921 | 4,802 | 0.151 | 3,180,983 | 3,313 | 0.104 | 0.047 | (0.041,0.052) | 1.246 | (1.192,1.302) | < 0.0001 | 1.251 |
|  | Female | 1,853,160 | 5,222 | 0.282 | 1,854,389 | 3,737 | 0.202 | 0.08 | (0.07,0.09) | 1.218 | (1.168,1.27) | < 0.0001 | 1.154 |
|  | Male | 1,201,774 | 1,953 | 0.163 | 1,202,364 | 1,516 | 0.126 | 0.036 | (0.027,0.046) | 1.148 | (1.073,1.228) | < 0.0001 | 7.351 |
|  | Black* | 593,883 | 1,261 | 0.212 | 594,121 | 734 | 0.124 | 0.089 | (0.074,0.104% | 1.436 | (1.311,1.573) | < 0.0001 | 5.914 |
|  | White | 2,038,526 | 5,117 | 0.251 | 2,039,791 | 4,042 | 0.198 | 0.053 | (0.044,0.062) | 1.198 | (1.15,1.249) | < 0.0001 | 2.534 |
| **Localized scleroderma [morphea]** | Primary | 3,180,579 | 4,034 | 0.127 | 3,182,140 | 2,718 | 0.085 | 0.041 | (0.036,0.046) | 1.295 | (1.234,1.36) | < 0.0001 | 3.068 |
|  | S1 | 1,072,745 | 1,767 | 0.165 | 1,072,725 | 1,878 | 0.175 | -0.01 | (-0.021,0.001) | 0.889 | (0.833,0.949) | 0.0004 | 0.658 |
|  | S2 | 3,180,270 | 2,670 | 0.084 | 3,181,896 | 1,786 | 0.056 | 0.028 | (0.024,0.032) | 1.286 | (1.211,1.365) | < 0.0001 | 0.585 |
|  | Female | 1,853,211 | 3,120 | 0.168 | 1,854,373 | 2,246 | 0.121 | 0.047 | (0.039,0.055) | 1.213 | (1.149,1.281) | < 0.0001 | 2.353 |
|  | Male | 1,203,010 | 302 | 0.025 | 1,203,136 | 22 | 0.018 | 0.007 | (0.004,0.011) | 1.266 | (1.062,1.509) | 0.0084 | 0.213 |
|  | Black* | 594,335 | 417 | 0.07 | 594,455 | 251 | 0.042 | 0.028 | (0.019,0.036) | 1.407 | (1.203,1.645) | < 0.0001 | 2.782 |
|  | White | 2,039,148 | 2,606 | 0.128 | 2,040,199 | 1,927 | 0.094 | 0.033 | (0.027,0.04) | 1.272 | (1.2,1.35) | < 0.0001 | 6.171 |
| **Urticaria** | Primary | 3,022,593 | 106,465 | 3.522 | 3,061,781 | 55,288 | 1.806 | 1.717 | (1.691,1.742) | 1.724 | (1.706,1.741) | < 0.0001 | 27.893^8^ |
|  | S1 | 1,043,681 | 49,997 | 4.79 | 1,051,888 | 36,963 | 3.514 | 1.276 | (1.222,1.33) | 1.297 | (1.279,1.314) | < 0.0001 | 24.336 |
|  | S2 | 3,136,474 | 69,317 | 2.21 | 3,181,192 | 36,782 | 1.156 | 1.054 | (1.034,1.074) | 1.662 | (1.641,1.683) | < 0.0001 | 7.27 |
|  | Female | 1,800,025 | 79,151 | 4.397 | 1,827,401 | 41,231 | 2.256 | 2.141 | (2.104,2.178) | 1.724 | (1.704,1.745) | < 0.0001 | 27.723 |
|  | Male | 1,180,371 | 28,133 | 2.383 | 1,191,887 | 14,977 | 1.257 | 1.127 | (1.093,1.161) | 1.703 | (1.67,1.738) | < 0.0001 | 1.528 |
|  | Black | 572,740 | 18,269 | 3.19 | 578,483 | 8,621 | 1.49 | 1.699 | (1.644,1.755) | 1.787 | (1.741,1.833) | < 0.0001 | 12.392 |
|  | White | 1,3983,606 | 77,628 | 3.913 | 2,011,870 | 41,231 | 2.049 | 1.864 | (1.831,1.897) | 1.825 | (1.803,1.847) | < 0.0001 | 12.306 |
| **Lichen sclerosus** | Primary | 3,094,794 | 9,711 | 0.314 | 3,097,325 | 6,565 | 0.212 | 0.102 | (0.094,0.11) | 1.292 | (1.252,1.333) | < 0.0001 | 0.122 |
|  | S1 | 1,074,269 | 3,717 | 0.346 | 1,074,355 | 3,288 | 0.306 | 0.04 | (0.025,0.055) | 1.07 | (1.021,1.122) | 0.0046 | 0.195 |
|  | S2 | 3,218,513 | 6,514 | 0.202 | 3,221,551 | 4,339 | 0.135 | 0.068 | (0.061,0.074) | 1.298 | (1.249,1.349) | < 0.0001 | 0.045 |
|  | Female | 1,849,411 | 9,120 | 0.493 | 1,851,756 | 6,442 | 0.348 | 0.145 | (0.132,0.158) | 1.236 | (1.197,1.276) | < 0.0001 | 3.601 |
|  | Male | 1,202,503 | 444 | 0.037 | 1,202,683 | 254 | 0.021 | 0.016 | (0.011,0.02) | 1.563 | (1.34,1.824) | < 0.0001 | 5.199 |
|  | Black | 583,591 | 945 | 0.162 | 583,750 | 478 | 0.082 | 0.08 | (0.067,0.093) | 1.635 | (1.465,1.825) | < 0.0001 | 5.187 |
|  | White | 2,035,741 | 7,633 | 0.375 | 2,037,815 | 5,586 | 0.274 | 0.101 | (0.09,0.112) | 1.294 | (1.25,1.339) | < 0.0001 | 0.002 |
| **Cutaneous lupus** | Primary | 3,093,419 | 9,870 | 0.319 | 3,096,454 | 4,421 | 0.143 | 0.176 | (0.169,0.184) | 1.959 | (1.891,2.03) | < 0.0001 | 1.584 |
|  | S1 | 1,073,185 | 4,871 | 0.454 | 1,073,374 | 3,885 | 0.362 | 0.092 | (0.075,0.109) | 1.184 | (1.135,1.235) | < 0.0001 | 9.334 |
|  | S2 | 3,217,233 | 6,286 | 0.195 | 3,220,867 | 2,830 | 0.088 | 0.108 | (0.102,0.113) | 1.926 | (1.842,2.013) | < 0.0001 | 2.059 |
|  | Female | 1,848,979 | 8,321 | 0.45 | 1,851,546 | 3,647 | 0.197 | 0.253 | (0.241,0.265) | 2.003 | (1.926,2.082) | < 0.0001 | 2.091 |
|  | Male | 1,201,675 | 1,487 | 0.124 | 1,202,110 | 707 | 0.059 | 0.065 | (0.057,0.073) | 1.88 | (1.719,2.056) | < 0.0001 | 0.389 |
|  | Black | 581,908 | 2,406 | 0.413 | 582,468 | 1,282 | 0.22 | 0.193 | (0.173,0.214) | 1.566 | (1.464,1.676) | < 0.0001 | 2.282 |
|  | White | 2,036,438 | 6,480 | 0.318 | 2,038,278 | 2,985 | 0.146 | 0.172 | (0.162,0.181) | 2.054 | (1.967,2.145) | < 0.0001 | 0.516 |
| **Pemphigus diseses** | Primary | 3,101,409 | 519 | 0.017 | 3,101,514 | 282 | 0.009 | 0.008 | (0.006,0.009) | 1.614 | (1.396,1.866) | < 0.0001 | 8.601 |
|  | S1 | 1,075,819 | 206 | 0.019 | 1,075,823 | 203 | 0.019 | 0 | (-0.003,0.004) | 0.962 | (0.792,1.167) | 0.6918 | 0.268 |
|  | S2 | 3,226,340 | 319 | 0.01 | 3,226,460 | 165 | 0.005 | 0.005 | (0.003,0.006) | 1.673 | (1.387,2.019) | < 0.0001 | 1.751 |
|  | Female | 1,855,744 | 337 | 0.018 | 1,855,830 | 175 | 0.009 | 0.009 | (0.006,0.011) | 1.688 | (1.406,2.026) | < 0.0001 | 2.729 |
|  | Male | 1,202,707 | 166 | 0.014 | 1,202,734 | 94 | 0.008 | 0.006 | (0.003,0.009) | 1.574 | (1.222,2.027) | 0.0004 | 3.656 |
|  | Black | 584,025 | 91 | 0.016 | 584,030 | 43 | 0.007 | 0.008 | (0.004,0.012) | 1.766 | (1.229,2.539) | 0.0019 | 0.018 |
|  | White | 2,041,242 | 353 | 0.017 | 2,041,333 | 196 | 0.01 | 0.008 | (0.005,0.01) | 1.704 | (1.431,2.029) | < 0.0001 | 11.05 |
| **Pemphigoid diseases** | Primary | 3,100,837 | 1,805 | 0.058 | 3,101,124 | 975 | 0.031 | 0.027 | (0.023,0.03) | 1.62 | (1.498,1.751) | < 0.0001 | 6.117 |
|  | S1 | 1,075,699 | 714 | 0.066 | 1,075,644 | 680 | 0.063 | 0.003 | (-0.004,0.01) | 0.994 | (0.895,1.104) | 0.9132 | 0.002 |
|  | S2 | 3,225,632 | 1,093 | 0.034 | 3,226,005 | 600 | 0.019 | 0.015 | (0.013,0.018) | 1.575 | (1.426,1.74) | < 0.0001 | 1.639 |
|  | Female | 1,855,377 | 1,115 | 0.06 | 1,855,568 | 542 | 0.029 | 0.031 | (0.027,0.035) | 1.797 | (1.622,1.991) | < 0.0001 | 3.335 |
|  | Male | 1,202,504 | 671 | 0.056 | 1,202,574 | 423 | 0.035 | 0.021 | (0.015,0.026) | 1.417 | (1.254,1.6) | < 0.0001 | 0.776 |
|  | Black | 583,963 | 275 | 0.047 | 583,991 | 140 | 0.024 | 0.023 | (0.016,0.03) | 1.617 | (1.319,1.982) | < 0.0001 | 0.3 |
|  | White | 2,040,773 | 1,316 | 0.064 | 2,040,973 | 786 | 0.039 | 0.026 | (0.022,0.03) | 1.59 | (1.456,1.737) | < 0.0001 | 7.214 |
| **Dermatitis herpetiformis** | Primary | 3,101,217 | 675 | 0.022 | 3,101,436 | 387 | 0.012 | 0.009 | (0.007,0.011) | 1.526 | (1.346,1.729) | < 0.0001 | 2.608 |
|  | S1 | 1,075,811 | 301 | 0.028 | 1,075,782 | 306 | 0.028 | 0 | (-0.005,0.004) | 0.931 | (0.794,1.091) | 0.3773 | 0.411 |
|  | S2 | 3,226,144 | 438 | 0.014 | 3,226,384 | 289 | 0.009 | 0.005 | (0.003,0.006) | 1.309 | (1.129,1.519) | 0.0004 | 0.596 |
|  | Female | 1,855,635 | 445 | 0.024 | 1,855,768 | 260 | 0.014 | 0.01 | (0.007,0.013) | 1.496 | (1.284,1.743) | < 0.0001 | 3.333 |
|  | Male | 1,202,647 | 223 | 0.019 | 1,202,694 | 141 | 0.012 | 0.007 | (0.004,0.01) | 1.411 | (1.143,1.742) | 0.0013 | 0.101 |
|  | Black | 584,056 | 45 | 0.008 | 584,067 | 21 | 0.004 | 0.004 | (0.001,0.007) | 1.765 | (1.051,2.962) | 0.0295 | 6.888 |
|  | White | 2,041,024 | 549 | 0.027 | 2,041,176 | 342 | 0.017 | 0.01 | (0.007,0.013) | 1.519 | (1.327,1.738) | < 0.0001 | 6.843 |
| **Systemic lupus erythematosus (SLE)** | Primary | 3,083,416 | 12,070 | 0.391 | 3,089,170 | 6,492 | 0.21 | 0.181 | (0.173,0.19) | 1.632 | (1.584,1.682) | < 0.0001 | 50.322^9^ |
|  | S1 | 1,070,934 | 6,076 | 0.567 | 1,071,124 | 5,120 | 0.478 | 0.089 | (0.07,0.109) | 1.121 | (1.08,1.164) | < 0.0001 | 4.608 |
|  | S2 | 3,206,817 | 7,817 | 0.244 | 3,213,245 | 4,299 | 0.134 | 0.11 | (0.103,0.117) | 1.579 | (1.521,1.639) | < 0.0001 | 9.621 |
|  | Female | 1,839,892 | 10,107 | 0.549 | 1,845,122 | 5,584 | 0.303 | 0.247 | (0.233,0.26) | 1.591 | (1.54,1.644) | < 0.0001 | 39.397 |
|  | Male | 1,201,055 | 1,658 | 0.138 | 1,201,599 | 928 | 0.077 | 0.061 | (0.053,0.069) | 1.591 | (1.468,1.724) | < 0.0001 | 9.803 |
|  | Black | 572,617 | 3,190 | 0.551 | 579,625 | 2,093 | 0.361 | 0.19 | (0.166,0.215) | 1.275 | (1.207,1.347) | < 0.0001 | 38.499 |
|  | White | 2,031,504 | 7,129 | 0.351 | 2,034,224 | 4,182 | 0.206 | 0.145 | (0.135,0.156) | 1.608 | (1.548,1.671) | < 0.0001 | 3.534 |
| **Systemic sclerosis** | Primary | 3,098,647 | 3,399 | 0.11 | 3,098,538 | 2,089 | 0.067 | 0.042 | (0.038,0.047) | 1.425 | (1.35,1.505) | < 0.0001 | 8.238 |
|  | S1 | 1,075,127 | 1,509 | 0.14 | 1,074,388 | 1,845 | 0.172 | -0.031 | (-0.042,-0.021) | 0.772 | (0.721,0.826) | < 0.0001 | 0.065 |
|  | S2 | 3,223,233 | 2,115 | 0.066 | 3,223,215 | 1,369 | 0.042 | 0.023 | (0.02,0.027) | 1.338 | (1.25,1.432) | < 0.0001 | 4.223 |
|  | Female | 1,853,329 | 2,725 | 0.147 | 1,853,212 | 1,753 | 0.095 | 0.052 | (0.045,0.06) | 1.362 | (1.283,1.446) | < 0.0001 | 8.445 |
|  | Male | 1,202,399 | 630 | 0.052 | 1,202,394 | 376 | 0.031 | 0.021 | (0.016,0.026) | 1.491 | (1.312,1.694) | < 0.0001 | 0.374 |
|  | Black | 583,507 | 648 | 0.111 | 583,430 | 402 | 0.069 | 0.042 | (0.031,0.053) | 1.346 | (1.188,1.524) | < 0.0001 | 4.461 |
|  | White | 2,039,355 | 2,295 | 0.113 | 2,039,186 | 1,555 | 0.076 | 0.036 | (0.03,0.042) | 1.394 | (1.307,1.487) | < 0.0001 | 4.597 |
| **Rheumatoid arthritis** | Primary | 3,042,930 | 51,812 | 1.703 | 3,062,668 | 27,248 | 0.89 | 0.813 | (0.795,0.831) | 1.679 | (1.655,1.704) | < 0.0001 | 48.559^10^ |
|  | S1 | 1,058,907 | 22,583 | 2.133 | 1,060,348 | 16,768 | 1.581 | 0.551 | (0.515,0.588) | 1.279 | (1.253,1.305) | < 0.0001 | 24.111 |
|  | S2 | 3,161,790 | 34,040 | 1.077 | 3,183,367 | 17,852 | 0.561 | 0.516 | (0.502,0.53) | 1.665 | (1.635,1.695) | < 0.0001 | 25.336 |
|  | Female | 1,812,976 | 37,171 | 2.05 | 1,826,964 | 19,738 | 1.08 | 0.97 | (0.944,0.995) | 1.666 | (1.638,1.695) | < 0.0001 | 29.237 |
|  | Male | 1,188,862 | 13,007 | 1.094 | 1,193,554 | 7,237 | 0.606 | 0.488 | (0.464,0.511) | 1.611 | (1.565,1.658) | < 0.0001 | 1.904 |
|  | Black | 574,825 | 9,433 | 1.641 | 577,428 | 4,817 | 0.834 | 0.807 | (0.766,0.847) | 1.638 | (1.582,1.696) | < 0.0001 | 10.553 |
|  | White | 2,000,088 | 35,156 | 1.758 | 2,013,236 | 19,998 | 0.993 | 0.764 | (0.742,0.787) | 1.676 | (1.647,1.705) | < 0.0001 | 28.864 |
| **Sjögren syndrome** | Primary* | 3,517,075 | 19,760 | 0.562 | 3,521,382 | 14,455 | 0.41 | 0.151 | (0.141,0.162) | 1.206 | (1.181,1.233) | < 0.0001 | 0.007 |
|  | S1 | 1,070,105 | 6,916 | 0.646 | 1,069,335 | 8,393 | 0.785 | -0.139 | (-0.161,-0.116) | 0.778 | (0.754,0.804) | < 0.0001 | 2.406 |
|  | S2 | 3,210,788 | 10,622 | 0.331 | 3,214,749 | 7,813 | 0.243 | 0.088 | (0.08,0.096) | 1.177 | (1.143,1.212) | < 0.0001 | 4.254 |
|  | Female | 1,843,550 | 14,529 | 0.788 | 1,846,706 | 10,846 | 0.587 | 0.201 | (0.184,0.218) | 1.167 | (1.138,1.196) | < 0.0001 | 29.237 |
|  | Male | 1,201,349 | 2,658 | 0.221 | 1,201,846 | 2,011 | 0.167 | 0.054 | (0.043,0.065) | 1.183 | (1.117,1.254) | < 0.0001 | 5.826 |
|  | Black | 580,296 | 2,691 | 0.464 | 580,945 | 1,412 | 0.243 | 0.221 | (0.199,0.242) | 1.567 | (1.469,1.671) | < 0.0001 | 1.978 |
|  | White | 2,032,346 | 11,496 | 0.566 | 2,034,697 | 9,118 | 0.448 | 0.118 | (0.104,0.131) | 1.194 | (1.162,1.227) | < 0.0001 | 0.213 |
| **Ankylosing spondylitis** | Primary* | 3,526,827 | 6,441 | 0.183 | 3,528,428 | 3,398 | 0.096 | 0.086 | (0.081,0.092) | 1.67 | (1.602,1.74) | < 0.0001 | 36.158^11^ |
|  | S1 | 1,071,736 | 2,059 | 0.192 | 1,071,801 | 1,536 | 0.143 | 0.049 | (0.038,0.06) | 1.265 | (1.184,1.351) | < 0.0001 | 3.791 |
|  | S2 | 3,220,729 | 3,445 | 0.107 | 3,222,234 | 1,811 | 0.056 | 0.051 | (0.046,0.055) | 1.647 | (1.556,1.743) | < 0.0001 | 0.344 |
|  | Female | 1,852,653 | 3,103 | 0.167 | 1,853,437 | 1,565 | 0.084 | 0.083 | (0.076,0.09) | 1.73 | (1.628,1.838) | < 0.0001 | 12.108 |
|  | Male | 1,200,769 | 1,966 | 0.164 | 1,201,292 | 1,054 | 0.088 | 0.076 | (0.067,0.085) | 1.662 | (1.542,1.791) | < 0.0001 | 6.287 |
|  | Black | 581,804 | 623 | 0.107 | 681,926 | 289 | 0.05 | 0.057 | (0.047,0.068) | 1.768 | (1.537,2.033) | < 0.0001 | 3.076 |
|  | White | 2,038,015 | 3,871 | 0.19 | 2,039,139 | 2,099 | 0.103 | 0.087 | (0.08,0.094) | 1.74 | (1.65,1.835) | < 0.0001 | 18.076 |
| **Polyarteritis nodosa** | Primary* | 3,531,536 | 978 | 0.028 | 3,531,770 | 372 | 0.011 | 0.017 | (0.015,0.019) | 2.328 | (2.066,2.623 | < 0.0001 | 0.001 |
|  | S1 | 1,072,656 | 454 | 0.042 | 1,072,662 | 343 | 0.032 | 0.01 | (0.005,0.016) | 1.251 | (1.087,1.439) | 0.0017 | 0.104 |
|  | S2 | 3,225,057 | 515 | 0.016 | 3,225,4212 | 222 | 0.007 | 0.009 | (0.007,0.011) | 2.004 | (1.712,2.346) | < 0.0001 | 6.658 |
|  | Female | 1,854,643 | 568 | 0.031 | 1,854,784 | 226 | 0.012 | 0.018 | (0.015,0.021) | 2.21 | (1.894,2.579) | < 0.0001 | 0.003 |
|  | Male | 1,202,620 | 336 | 0.028 | 1,202,683 | 124 | 0.01 | 0.018 | (0.014,0.021) | 2.429 | (1.977,2.984) | < 0.0001 | 0.181 |
|  | Black | 582,151 | 125 | 0.021 | 582,177 | 47 | 0.008 | 0.013 | (0.009,0.018) | 2.235 | (1.598,3.127) | < 0.0001 | 0 |
|  | White | 2,041,104 | 716 | 0.035 | 2,041,278 | 277 | 0.014 | 0.022 | (0.018,0.025) | 2.446 | (2.129,2.81) | < 0.0001 | 0.072 |
| **Polymyalgia rheumatica** | Primary* | 3,523,567 | 10,289 | 0.292 | 3,526,270 | 7,297 | 0.207 | 0.085 | (0.078,0.092) | 1.242 | (1.205,1.28) | < 0.0001 | 4.933 |
|  | S1* | 2,660,196 | 8,646 | 0.325 | 2,658,683 | 7,614 | 0.286 | 0.039 | (0.029,0.048) | 1.048 | (1.016,1.081) | 0.0029 | 1.208 |
|  | S2 | 3,219,047 | 9,030 | 0.281 | 3,221,424 | 6,321 | 0.196 | 0.084 | (0.077,0.092) | 1.254 | (1.214,1.295) | < 0.0001 | 11.056 |
|  | Female | 1,850,847 | 5,655 | 0.306 | 1,852,498 | 3,771 | 0.204 | 0.102 | (0.092,0.112) | 1.308 | (1.255,1.363) | < 0.0001 | 4.261 |
|  | Male | 1,201,101 | 2,626 | 0.219 | 1,201,649 | 2,212 | 0.184 | 0.035 | (0.023,0.046) | 1.06 | (1.002,1.122) | 0.0420 | 5.017 |
|  | Black | 581,842 | 1,040 | 0.179 | 582,092 | 482 | 0.083 | 0.096 | (0.083,0.109) | 1.784 | (1.601,1.988) | < 0.0001 | 3.129 |
|  | White | 2,036,418 | 6,461 | 0.317 | 2,038,184 | 5,052 | 0.248 | 0.069 | (0.059,0.08) | 1.208 | (1.164,1.253) | < 0.0001 | 2.754 |
| **Dermatomyositis** | Primary* | 3,529,761 | 2,567 | 0.073 | 3,530,818 | 1,413 | 0.04 | 0.033 | (0.029,0.036) | 1.602 | (1.501,1.71) | < 0.0001 | 2.138 |
|  | S1* | 2,662,266 | 1,939 | 0.073 | 2,662,213 | 1,569 | 0.059 | 0.014 | (0.01,0.018) | 1.148 | (1.074,1.227) | < 0.0001 | 0.15 |
|  | S2 | 3,224,448 | 2,433 | 0.075 | 3,225,537 | 1,347 | 0.042 | 0.034 | (0.03,0.037) | 1.59 | (1.488,1.699) | < 0.0001 | 2.756 |
|  | Female | 1,854,021 | 1,660 | 0.09 | 1,854,741 | 852 | 0.046 | 0.044 | (0.038,0.049) | 1.705 | (1.57,1.852) | < 0.0001 | 0.962 |
|  | Male | 1,202,771 | 666 | 0.055 | 1,203,072 | 386 | 0.032 | 0.023 | (0.018,0.029) | 1.536 | (1.355,1.742) | < 0.0001 | 0.027 |
|  | Black | 581,811 | 544 | 0.094 | 582,052 | 268 | 0.046 | 0.047 | (0.038,0.057) | 1.697 | (1.466,1.964) | < 0.0001 | 0.085 |
|  | White | 2,041,043 | 1,531 | 0.075 | 2,041,538 | 899 | 0.044 | 0.031 | (0.026,0.036) | 1.602 | (1.475,1.739) | < 0.0001 | 1.891 |
| **Sarcoidosis** | Primary* | 3,518,477 | 9,582 | 0.272 | 3,525,077 | 4,837 | 0.137 | 0.135 | (0.128,0.142) | 1.754 | (1.694,1.816) | < 0.0001 | 20.477^12^ |
|  | S1* | 2,658,017 | 8,181 | 0.308 | 2,659,606 | 4,502 | 0.169 | 0.139 | (0.13,0.147) | 1.689 | (1.629,1.752) | < 0.0001 | 1.827 |
|  | S2 | 3,214,189 | 8,667 | 0.27 | 3,220,187 | 4,433 | 0.138 | 0.132 | (0.125,0.139) | 1.726 | (1.665,1.79) | < 0.0001 | 16.846 |
|  | Female | 1,847,703 | 5,457 | 0.295 | 1,851,683 | 2,543 | 0.137 | 0.158 | (0.149,0.167) | 1.885 | (1.799,1.976) | < 0.0001 | 7.53 |
|  | Male | 1,199,414 | 2,734 | 0.228 | 1,201,165 | 1,573 | 0.131 | 0.097 | (0.086,0.108) | 1.553 | (1.46,1.653) | < 0.0001 | 0.226 |
|  | Black | 577,440 | 2,816 | 0.488 | 579,080 | 1,757 | 0.303 | 0.184 | (0.161,0.207) | 1.348 | (1.27,1.431) | < 0.0001 | 15.619 |
|  | White | 2,036,534 | 4,609 | 0.226 | 2,039,075 | 2,564 | 0.126 | 0.101 | (0.092,0.109) | 1.697 | (1.617,1.781) | < 0.0001 | 3.208 |
| **Systemic vasculits** | Primary* | 3,530,662 | 1,751 | 0.05 | 3,531,091 | 889 | 0.025 | 0.024 | (0.022,0.027) | 1.743 | (1.608,1.89) | < 0.0001 | 0.005 |
|  | S1 | 1,072,444 | 712 | 0.066 | 1,072,458 | 647 | 0.06 | 0.006 | (-0.001,0.013) | 1.041 | (0.936,1.158) | 0.4589 | 1.312 |
|  | S2 | 3,224,183 | 919 | 0.029 | 3,224,706 | 474 | 0.015 | 0.014 | (0.012,0.016) | 1.678 | (1.502,1.875) | < 0.0001 | 0.488 |
|  | Female | 1,854,187 | 997 | 0.054 | 1,854,392 | 457 | 0.025 | 0.029 | (0.025,0.033) | 1.914 | (1.713,2.138) | < 0.0001 | 0.517 |
|  | Male | 1,202,329 | 558 | 0.046 | 1,202,405 | 287 | 0.024 | 0.023 | (0.018,0.027) | 1.742 | (1.511,2.009) | < 0.0001 | 0.875 |
|  | Black | 582,077 | 219 | 0.038 | 582,128 | 90 | 0.015 | 0.022 | (0.016,0.028) | 2.033 | (1.59,2.599) | < 0.0001 | 0.02 |
|  | White | 2,040,540 | 1,204 | 0.059 | 2,040,739 | 630 | 0.031 | 0.028 | (0.024,0.032) | 1.808 | (1.642,1.991) | < 0.0001 | 0.053 |
| **Myasthenia gravis** | Primary* | 3,528,465 | 3,327 | 0.094 | 3,529,793 | 1,849 | 0.052 | 0.042 | (0.038,0.046) | 1.59 | (1.502,1.683) | < 0.0001 | 22.752^13^ |
|  | S1 | 1,071,950 | 1,228 | 0.115 | 1,072,021 | 1,034 | 0.096 | 0.018 | (0.009,0.027) | 1.123 | (1.034,1.22) | 0.0059 | 0.998 |
|  | S2 | 3,222,147 | 1,892 | 0.059 | 3,223,460 | 1,009 | 0.031 | 0.027 | (0.024,0.031) | 1.624 | (1.504,1.753) | < 0.0001 | 4.266 |
|  | Female | 1,853,128 | 1,701 | 0.092 | 1,853,743 | 941 | 0.051 | 0.041 | (0.036,0.046) | 1.581 | (1.46,1.712) | < 0.0001 | 8.09 |
|  | Male | 1,201,473 | 1,165 | 0.097 | 1,202,046 | 684 | 0.057 | 0.04 | (0.033,0.047) | 1.524 | (1.387,1.675) | < 0.0001 | 16.322 |
|  | Black | 581,742 | 355 | 0.061 | 581,874 | 207 | 0.036 | 0.025 | (0.017,0.033) | 1.427 | (1.202,1.694) | < 0.0001 | 1.048 |
|  | White | 2,039,163 | 2,227 | 0.109 | 2,039,986 | 1,290 | 0.063 | 0.046 | (0.04,0.052) | 1.635 | (1.526,1.751) | < 0.0001 | 21.217 |
| **Multiple sclerosis** | Primary* | 3,515,482 | 10,756 | 0.306 | 3,516,852 | 7,622 | 0.217 | 0.089 | (0.082,0.097) | 1.248 | (1.212,1.285) | < 0.0001 | 255.26^14^ |
|  | S1 | 1,068,411 | 4,977 | 0.466 | 1,067,663 | 4,384 | 0.411 | 0.055 | (0.037,0.073) | 1.074 | (1.031,1.119) | 0.0006 | 2.251 |
|  | S2 | 3,209,756 | 6,040 | 0.188 | 3,210,401 | 4,106 | 0.128 | 0.06 | (0.054,0.066) | 1.275 | (1.225,1.326) | < 0.0001 | 69.431 |
|  | Female | 1,843,800 | 7,116 | 0.386 | 1,844,656 | 5,203 | 0.282 | 0.104 | (0.092,0.116) | 1.201 | (1.158,1.244) | < 0.0001 | 139.189 |
|  | Male | 1,199,832 | 2,471 | 0.206 | 1,199,853 | 1,721 | 0.143 | 0.063 | (0.052,0.073) | 1.286 | (1.209,1.368) | < 0.0001 | 56.066 |
|  | Black | 579,746 | 1,501 | 0.259 | 579,709 | 1,216 | 0.21 | 0.049 | (0.032,0.067) | 1.025 | (0.95,1.106) | 0.5229 | 26.388 |
|  | White | 2,031,166 | 6,986 | 0.344 | 2,031,222 | 5,380 | 0.265 | 0.079 | (0.068,0.09) | 1.226 | (1.184,1.271) | < 0.0001 | 262.841 |
| **Neuromyelitis optica** | Primary* | 3,531,743 | 393 | 0.011 | 3,531,769 | 271 | 0.008 | 0.003 | (0.002,0.00%) | 1.279 | (1.096,1.493) | 0.0018 | 3.188 |
|  | S1 | 1,072,708 | 184 | 0.017 | 1,072,710 | 200 | 0.019 | -0.001 | (-0.005,0.002) | 0.873 | (0.715,1.067) | 0.1852 | 1.359 |
|  | S2 | 3,225,384 | 207 | 0.006 | 3,225,468 | 145 | 0.004 | 0.002 | (0.001,0.003) | 1.235 | (0.999,1.528) | 0.0505 | 0.535 |
|  | Female | 1,854,687 | 271 | 0.015 | 1,854,742 | 162 | 0.009 | 0.006 | (0.004,0.008) | 1.47 | (1.21,1.786) | < 0.0001 | 0.457 |
|  | Male | 1,202,779 | 65 | 0.005 | 1,202,780 | 47 | 0.004 | 0.001 | (0,0.003) | 1.231 | (0.845,1.791) | 0.2779 | 2.027 |
|  | Black | 582,072 | 123 | 0.021 | 582,086 | 74 | 0.013 | 0.008 | (0.004,0.013) | 1.389 | (1.041,1.853) | 0.0251 | 0.419 |
|  | White | 2,041,367 | 173 | 0.008 | 2,041,368 | 140 | 0.007 | 0.002 | (0,0.003) | 1.162 | (0.93,1.453) | 0.1852 | 1.366 |
| **Celiac disease** | Primary* | 3,519,339 | 12,276 | 0.349 | 3,521,101 | 9,355 | 0.266 | 0.083 | (0.075,0.091) | 1.157 | (1.126,1.189) | < 0.0001 | 22.59^15^ |
|  | S1 | 1,069,811 | 5,553 | 0.519 | 1,069,103 | 6,703 | 0.627 | -0.108 | (-0.128,-0.088) | 0.781 | (0.753,0.809) | < 0.0001 | 4.849 |
|  | S2 | 3,213,468 | 1,138 | 0.222 | 3,215,132 | 5,344 | 0.166 | 0.056 | (0.049,0.063) | 1.157 | (1.116,1.199) | < 0.0001 | 4.872 |
|  | Female | 1,847,156 | 1,847,156 | 0.435 | 1,847,948 | 5,937 | 0.321 | 0.114 | (0.101,0.126) | 1.185 | (1.146,1.225) | < 0.0001 | 4.467 |
|  | Male | 1,200,203 | 2,559 | 0.213 | 1,200,683 | 1,949 | 0.162 | 0.051 | (0.04,0.062) | 1.17 | (1.103,1.241) | < 0.0001 | 2.018 |
|  | Black | 581,752 | 809 | 0.139 | 581,840 | 476 | 0.082 | 0.057 | (0.045,0.069) | 1.409 | (1.258,1.578) | < 0.0001 | 0.053 |
|  | White | 2,032,537 | 8,664 | 0.426 | 2,033,697 | 6,522 | 0.321 | 0.106 | (0.094,0.117) | 1.252 | (1.213,1.293) | < 0.0001 | 20.783 |
| **Crohn's disease** | Primary* | 3,512,074 | 14,452 | 0.411 | 3,514,107 | 9,329 | 0.265 | 0.146 | (0.137,0.155) | 1.369 | (1.334,1.405) | < 0.0001 | 53.704^16^ |
|  | S1 | 1,067,932 | 6,071 | 0.568 | 1,066,846 | 6,205 | 0.582 | -0.013 | (-0.033,0.007) | 0.923 | (0.891,0.956) | < 0.0001 | 17.45 |
|  | S2 | 3,206,428 | 8,346 | 0.26 | 3,208,894 | 5,271 | 0.164 | 0.096 | (0.089,0.103) | 1.373 | (1.326,1.421) | < 0.0001 | 18.179 |
|  | Female | 1,844,376 | 8,402 | 0.456 | 1,845,788 | 4,918 | 0.266 | 0.189 | (0.177,0.201) | 1.499 | (1.447,1.553) | < 0.0001 | 33.811 |
|  | Male | 1,196,478 | 4,444 | 0.371 | 1,197,058 | 3,040 | 0.254 | 0.117 | (0.103,0.132) | 1.304 | (1.246,1.366) | < 0.0001 | 6.397 |
|  | Black | 580,216 | 1,833 | 0.316 | 580,392 | 1,032 | 0.178 | 0.138 | (0.12,0.156) | 1.476 | (1.367,1.593) | < 0.0001 | 1.34 |
|  | White | 2,027,987 | 9,951 | 0.491 | 2,029,445 | 6,398 | 0.315 | 0.175 | (0.163,0.188) | 1.469 | (1.424,1.516) | < 0.0001 | 47.742 |
| **Ulcerative colitis** | Primary* | 3,511,073 | 21,183 | 0.603 | 3,513,919 | 14,477 | 0.412 | 0.191 | (0.181,0.202) | 1.292 | (1.265,1.32) | < 0.0001 | 69.677^17^ |
|  | S1 | 1,068,762 | 6,848 | 0.641 | 1,067,601 | 6,954 | 0.651 | -0.011 | (-0.032,0.011) | 0.93 | (0.9,0.962) | < 0.0001 | 39.572 |
|  | S2 | 3,205,691 | 11,420 | 0.356 | 3,208,714 | 7,692 | 0.24 | 0.117 | (0.108,0.125) | 1.286 | (1.249,1.323) | < 0.0001 | 10.082 |
|  | Female | 1,845,032 | 10,625 | 0.576 | 1,846,373 | 7,077 | 0.383 | 0.193 | (0.178,0.207) | 1.313 | (1.274,1.353) | < 0.0001 | 34.174 |
|  | Male | 1,195,724 | 6,677 | 0.558 | 1,196,956 | 4,664 | 0.39 | 0.169 | (0.151,0.186) | 1.281 | 1.234,1.33 | < 0.0001 | 41.789 |
|  | Black | 580,369 | 2,399 | 0.413 | 580,700 | 1,480 | 0.255 | 0.158 | (0.138,0.179) | 1.34 | (1.256,1.43) | < 0.0001 | 6.622 |
|  | White | 2,028,066 | 12,978 | 0.64 | 2,029,822 | 9,381 | 0.462 | 0.178 | (0.163,0.192) | 1.309 | (1.275,1.345) | < 0.0001 | 75.103 |
| **Primary biliary cirrhosis** | Primary* | 3,530,395 | 2,908 | 0.082 | 3,531,010 | 1,461 | 0.041 | 0.041 | (0.037,0.045) | 1.76 | (1.653,1.87) | < 0.0001 | 9.071 |
|  | S1* | 2,662,619 | 1,992 | 0.075 | 2,662,286 | 1,866 | 0.07 | 0.005 | (0,0.009) | 0.988 | (0.928,1.053) | 0.7128 | 24.48 |
|  | S2 | 3,225,142 | 2,628 | 0.081 | 3,225,762 | 1,191 | 0.037 | 0.045 | (0.041,0.048) | 1.945 | (1.816,2.083) | < 0.0001 | 0.371 |
|  | Female | 1,854,342 | 1,870 | 0.101 | 1,854,789 | 913 | 0.049 | 0.052 | (0.046,0.057) | 1.794 | (1.657,1.941) | < 0.0001 | 0.171 |
|  | Male | 1,203,142 | 626 | 0.052 | 1,203,244 | 264 | 0.022 | 0.03 | (0.025,0.035) | 2.12 | (1.836,2.448) | < 0.0001 | 0.019 |
|  | Black | 582,273 | 300 | 0.052 | 582,324 | 148 | 0.025 | 0.026 | (0.019,0.033) | 1.672 | (1.373,2.036) | < 0.0001 | 0.101 |
|  | White | 2,041,123 | 1,940 | 0.095 | 2,041,607 | 979 | 0.048 | 0.047 | (0.042,0.052) | 1.873 | (1.735,2.023) | < 0.0001 | 0.064 |
| **Other chronic pancreatitis** | Primary | 3,225,979 | 11,705 | 0.363 | 3,225,877 | 7,468 | 0.232 | 0.131 | (0.123,0.14) | 1.372 | (1.333,1.412) | < 0.0001 | 65.35^18^ |
|  | S1 | 1,062,590 | 3,663 | 0.345 | 1,060,720 | 5,668 | 0.534 | -0.19 | (-0.207,-0.172) | 0.609 | (0.584,0.635) | < 0.0001 | 58.042 |
|  | S2 | 3,213,643 | 7,243 | 0.225 | 3,214,007 | 4,798 | 0.149 | 0.076 | (0.069,0.083) | 1.301 | (1.255,1.35) | < 0.0001 | 12.551 |
|  | Female | 1,712,780 | 5,629 | 0.329 | 1,712,928 | 3,379 | 0.197 | 0.131 | (0.121,0.142) | 1.435 | (1.375,1.497) | < 0.0001 | 37.285 |
|  | Male | 1,107,944 | 4,283 | 0.387 | 1,107,806 | 2,892 | 0.261 | 0.126 | (0.111,0.14) | 1.298 | (1.238,1.36) | < 0.0001 | 39.408 |
|  | Black | 576,797 | 1,894 | 0.328 | 576,374 | 1,568 | 0.272 | 0.056 | (0.036,0.076) | 1.003 | (0.938,1.072) | 0.9301 | 13.359 |
|  | White | 1,995,586 | 7,393 | 0.37 | 1,995,688 | 4,874 | 0.244 | 0.126 | (0.115,0.137) | 1.432 | (1.381,1.485) | < 0.0001 | 49.469 |
| **Autoimmune hepatitis** | Primary* | 3,529,605 | 4,319 | 0.122 | 3,530,833 | 1,756 | 0.05 | 0.073 | (0.068,0.077) | 2.18 | (2.062,2.304) | < 0.0001 | 0.772 |
|  | S1* | 2,662,335 | 3,076 | 0.116 | 2,662,237 | 2,173 | 0.082 | 0.034 | (0.029,0.039) | 1.31 | (1.24,1.384) | < 0.0001 | 32.457 |
|  | S2 | 3,224,402 | 3,986 | 0.124 | 3,225,531 | 1,538 | 0.048 | 0.076 | (0.071,0.08) | 2.291 | (2.16,2.43) | < 0.0001 | 2.025 |
|  | Female | 1,853,779 | 2,776 | 0.15 | 1,854,637 | 1,064 | 0.057 | 0.092 | (0.086,0.099) | 2.29 | (2.134,2.458) | < 0.0001 | 0.157 |
|  | Male | 1,202,922 | 1,104 | 0.092 | 1,203,156 | 411 | 0.034 | 0.058 | (0.051,0.064) | 2.411 | (2.153,2.7) | < 0.0001 | 0.132 |
|  | Black | 582,053 | 552 | 0.095 | 582,155 | 263 | 0.045 | 0.05 | (0.04,0.059) | 1.747 | (1.508,2.023) | < 0.0001 | 0.082 |
|  | White | 2,040,773 | 2,838 | 0.139 | 2,041,473 | 1,127 | 0.055 | 0.084 | (0.078,0.09) | 2.385 | (2.226,2.555) | < 0.0001 | 2.746 |
| **Vitamin B12 deficiency anemia due to intrinsic factor deficiency** | Primary* | 3,524,837 | 8,382 | 0.238 | 3,527,617 | 4,848 | 0.137 | 0.1 | (0.094,0.107) | 1.522 | (1.47,1.577 | < 0.0001 | 22.24^19^ |
|  | S1* | 2,660,465 | 6,683 | 0.251 | 2,659,722 | 5,771 | 0.217 | 0.034 | (0.026,0.042) | 1.071 | (1.034,1.11) | 0.0001 | 12.804 |
|  | S2 | 3,220,112 | 7,517 | 0.233 | 3,222,395 | 4,392 | 0.136 | 0.097 | (0.091,0.104) | 1.502 | (1.447,1.559) | < 0.0001 | 23.738 |
|  | Female | 1,851,322 | 4,912 | 0.265 | 1,852,876 | 2,623 | 0.142 | 0.124 | (0.115,0.13) | 1.634 | (1.558,1.714) | < 0.0001 | 6.978 |
|  | Male | 1,201,811 | 1,814 | 0.151 | 1,202,234 | 1,223 | 0.102 | 0.049 | (0.04,0.058) | 1.322 | (1.229,1.421) | < 0.0001 | 1.461 |
|  | Black | 581,572 | 1,212 | 0.208 | 681,872 | 609 | 0.105 | 0.104 | (0.089,0.118) | 1.631 | (1.479,1.798) | < 0.0001 | 3.906 |
|  | White | 2,037,940 | 4,559 | 0.224 | 2,039,163 | 2,916 | 0.143 | 0.081 | (0.072,0.089) | 1.471 | (1.405,1.541) | < 0.0001 | 12.564 |
| **Other autoimmune hemolytic anemias** | Primary* | 3,529,943 | 6,322 | 0.179 | 3,531,239 | 2,388 | 0.068 | 0.111 | (0.106,0.117) | 2.348 | (2.24,2.462) | < 0.0001 | 5.517 |
|  | S1* | 2,662,363 | 4,116 | 0.155 | 2,662,300 | 3,072 | 0.115 | 0.039 | (0.033,0.045) | 1.24 | (1.184,1.3) | < 0.0001 | 0.022 |
|  | S2 | 3,224,587 | 6,114 | 0.19 | 3,225,816 | 2,265 | 0.07 | 0.119 | (0.114,0.125) | 2.388 | (2.275,2.506) | < 0.0001 | 1.622 |
|  | Female | 1,854,449 | 3,383 | 0.182 | 1,855,115 | 1,248 | 0.067 | 0.115 | (0.108,0.122) | 2.381 | (2.231,2.54) | < 0.0001 | 0.427 |
|  | Male | 1,202,434 | 2,597 | 0.216 | 1,202,982 | 917 | 0.076 | 0.14 | (0.13,0.149) | 2.543 | (2.359,2.742) | < 0.0001 | 1.621 |
|  | Black | 582,097 | 873 | 0.15 | 582,262 | 355 | 0.061 | 0.089 | (0.077,0.101) | 2.032 | (1.796,2.299) | < 0.0001 | 0.009 |
|  | White | 2,040,798 | 4,657 | 0.228 | 2,041,667 | 1,697 | 0.083 | 0.145 | (0.137,0.153) | 2.61 | (2.469,2.759) | < 0.0001 | 0.341 |
| **Immune thrombocytopenic purpura** | Primary* | 3,527,844 | 5,248 | 0.149 | 3,529,415 | 3,340 | 0.095 | 0.054 | (0.049,0.059) | 1.385 | (1.327,1.447) | < 0.0001 | 18.227^20^ |
|  | S1* | 2,661,559 | 3,923 | 0.147 | 2,661,242 | 3,811 | 0.143 | 0.004 | (-0.002,0.011) | 0.954 | (0.912,0.997) | 0.0381 | 2.483 |
|  | S2 | 3,222,894 | 4,651 | 0.144 | 3,224,276 | 2,864 | 0.089 | 0.055 | (0.05,0.061) | 1.428 | (1.363,1.496) | < 0.0001 | 19.802 |
|  | Female | 1,853,372 | 2,543 | 0.137 | 1,854,217 | 1,592 | 0.086 | 0.051 | (0.045,0.058) | 1.397 | (1.312,1.487) | < 0.0001 | 6.516 |
|  | Male | 1,202,007 | 1,868 | 0.155 | 1,202,489 | 1,110 | 0.092 | 0.063 | (0.054,0.072) | 1.502 | (1.395,1.618) | < 0.0001 | 9.444 |
|  | Black | 581,880 | 699 | 0.12 | 582,017 | 459 | 0.079 | 0.041 | (0.03,0.053) | 1.267 | (1.126,1.425) | < 0.0001 | 1.619 |
|  | White | 2,039,743 | 3,153 | 0.155 | 2,040,677 | 2,054 | 0.101 | 0.054 | (0.047,0.061) | 1.448 | (1.37,1.531) | < 0.0001 | 18.44 |
| **Antiphospholipid syndrome** | Primary | 3,225017 | 10,706 | 0.332 | 3,229,028 | 4,383 | 0.136 | 0.196 | (0.189,0.204) | 2.148 | (2.074,2.225) | < 0.0001 | 18.783^21^ |
|  | S1 | 1,062,005 | 5,032 | 0.474 | 1,062,399 | 3,607 | 0.34 | 0.134 | (0.117,0.151) | 1.319 | (1.264,1.376) | < 0.0001 | 12.365 |
|  | S2 | 3,212,535 | 6.430 | 0.2 | 3,217,363 | 2,799 | 0.087 | 0.113 | (0.107,0.119) | 1.984 | (1.898,2.074) | < 0.0001 | 2.725 |
|  | Female | 1,711,288 | 6,490 | 0.379 | 1,713,596 | 2,543 | 0.148 | 0.231 | (0.22,0.242) | 2.211 | (2.112,2.314) | < 0.0001 | 21.516 |
|  | Male | 1,108,965 | 2,878 | 0.26 | 1,110,013 | 1,178 | 0.106 | 0.153 | (0.142,0.165) | 2.148 | (2.007,2.299) | < 0.0001 | 2.319 |
|  | Black | 576,984 | 1,464 | 0.254 | 577,439 | 615 | 0.107 | 0.147 | (0.132,0.163) | 1.998 | (1.818,2.196) | < 0.0001 | 3.319 |
|  | White | 1,995,290 | 7,066 | 0.354 | 1,997,639 | 3,063 | 0.153 | 0.201 | (0.191,0.211) | 2.19 | (2.099,2.285) | < 0.0001 | 19.176 |
| **Autoimmune thyroiditis** | Primary | 3,205,992 | 28,868 | 0.9 | 3,216,932 | 17,576 | 0.546 | 0.354 | (0.341,0.367) | 1.44 | (1.414,1.468) | < 0.0001 | 2.913 |
|  | S1 | 1,058,126 | 10,790 | 1.02 | 1,059,153 | 9,074 | 0.857 | 0.163 | (0.137,0.189) | 1.123 | (1.092,1.155) | < 0.0001 | 7.063 |
|  | S2 | 3,192,084 | 19,182 | 0.601 | 3,203,990 | 11,219 | 0.35 | 0.251 | (0.24,0.261) | 1.479 | (1.445,1.514) | < 0.0001 | 0.389 |
|  | Female | 1,695,844 | 21,443 | 1.264 | 1,703,322 | 13,454 | 0.79 | 0.475 | (0.453,0.496) | 1.377 | (1.348,1.407) | < 0.0001 | 5.746 |
|  | Male | 1,108,212 | 3,992 | 0.36 | 1,109,697 | 2,175 | 0.196 | 0.164 | (0.15,0.178) | 1.603 | (1.521,1.689) | < 0.0001 | 0.112 |
|  | Black | 576,735 | 2,141 | 0.371 | 577,286 | 1,109 | 0.192 | 0.179 | (0.16,0.198) | 1.614 | (1.501,1.735) | < 0.0001 | 2.118 |
|  | White | 1,980,561 | 20,202 | 1.02 | 1,988,522 | 12,668 | 0.637 | 0.383 | (0.365,0.401) | 1.509 | (1.476,1.543) | < 0.0001 | 0.009 |
| **Type 1 diabetes mellitus** | Primary | 3,163,342 | 70,793 | 2.238 | 3,202,602 | 17,906 | 0.559 | 1.679 | (1.661,1.697) | 3.553 | (3.495,3.611) | < 0.0001 | 130.874^22^ |
|  | S1 | 1,044,375 | 23,234 | 2.32 | 1,049,474 | 11,144 | 1.062 | 1.259 | (1.224,1.293) | 2.086 | (2.04,2.134) | < 0.0001 | 47.053 |
|  | S2 | 3,145,535 | 45,710 | 1.453 | 3,189,703 | 11,680 | 0.366 | 1.087 | (1.072,1.102) | 3.449 | (3.379,3.52) | < 0.0001 | 48.993 |
|  | Female | 1,680,575 | 37,443 | 2.228 | 1,702,649 | 8,098 | 0.476 | 1.752 | (1.728,1.777) | 4.088 | (3.991,4.188) | < 0.0001 | 38.039 |
|  | Male | 1,085,658 | 24,343 | 2.242 | 1,098,609 | 7,169 | 0.653 | 1.59 | (1.558,1.621) | 3.051 | (2.971,3.132) | < 0.0001 | 42.715 |
|  | Black | 565,279 | 13,515 | 2.391 | 571,344 | 4,308 | 0.754 | 1.637 | (1.591,1.683) | 2.695 | (2.604,2.789) | < 0.0001 | 6.008 |
|  | White | 1,958,274 | 43,042 | 2.198 | 1,981,534 | 11,200 | 0.565 | 1.633 | (1.61,1.656) | 3.721 | (3.645,3.8) | < 0.0001 | 106.588 |
| **Chronic sinusitis** | Primary* | 3,301,941 | 226,308 | 6.854 | 3,410,229 | 142,989 | 4.193 | 2.661 | (2.626,2.695) | 1.451 | (1.441,1.461) | < 0.0001 | 430.327^23^ |
|  | S1* | 2,565,753 | 191,100 | 7.448 | 2,585,598 | 134,349 | 5.196 | 1.347 | (1.338,1.356) | 1.347 | (1.338,1.356) | < 0.0001 | 4.182 |
|  | S2 | 3,017,025 | 206,293 | 6.838 | 3,114,917 | 130,766 | 4.198 | 2.64 | (2.603,2.676) | 1.444 | (1.434,1.454) | < 0.0001 | 324.259 |
|  | Female | 1,721,754 | 134,386 | 7.805 | 1,784,436 | 83,319 | 4.669 | 3.136 | (3.085,3.187) | 1.479 | (1.466,1.492) | < 0.0001 | 242.733 |
|  | Male | 1,135,109 | 62,290 | 5.488 | 1,167,066 | 42,051 | 3.603 | 1.884 | (1.831,1.938) | 1.362 | (1.345,1.379) | < 0.0001 | 50.739 |
|  | Black | 557,567 | 32,130 | 5.763 | 569,549 | 16,536 | 2.903 | 2.859 | (2.784,2.934) | 1.679 | (1.648,1.711) | < 0.0001 | 103.503 |
|  | White | 1,884,710 | 143,128 | 7.594 | 1,956,011 | 98,489 | 5.035 | 2.559 | (2.51,2.608) | 1.432 | (1.421,1.444) | < 0.0001 | 307.307 |
| **Vasomotor and allergic rhinitis** | Primary* | 3,012,633 | 394,818 | 13.105 | 3,256,166 | 242,307 | 7.441 | 5.664 | (5.616,5.712) | 1.59 | (1.582,1.598) | < 0.0001 | 152.888^24^ |
|  | S1* | 2,422,565 | 333,027 | 13.747 | 2,448,627 | 251,590 | 10.275 | 3.472 | (3.414,3.53) | 1.266 | (1.26,1.273) | < 0.0001 | 1.526 |
|  | S2 | 2,747,793 | 355,505 | 12.938 | 2,969,532 | 219,847 | 7.403 | 5.534 | (5.485,5.584) | 1.575 | (1.567,1.584) | < 0.0001 | 76.949 |
|  | Female | 1,562,282 | 228,460 | 14.623 | 1,695,336 | 138,929 | 8.195 | 6.429 | (6.36,6.498) | 1.613 | (1.602,1.624) | < 0.0001 | 81.902 |
|  | Male | 1,039,524 | 111,214 | 10.699 | 1,118,395 | 72,255 | 6.461 | 4.238 | (4.163,4.313) | 1.497 | (1.483,1.511) | < 0.0001 | 3.227 |
|  | Black | 494,078 | 75,074 | 15.195 | 535,489 | 43,047 | 8.039 | 7.156 | (7.032,7.28) | 1.655 | (1.636,1.675) | < 0.0001 | 35.551 |
|  | White | 1,733,902 | 216,951 | 12.512 | 1,870,008 | 146,052 | 7.81 | 4.702 | (4.64,4.765) | 1.539 | (1.529,1.549) | < 0.0001 | 5.785 |
| **Asthma** | Primary | 2,803,198 | 251,089 | 8.957 | 3,017,831 | 129,146 | 4.279 | 4.678 | (4.637,4.718) | 1.892 | (1.879,1.905) | < 0.0001 | 114.856^25^ |
|  | S1 | 945,318 | 113,370 | 11.993 | 980,527 | 70,962 | 7.237 | 4.756 | (4.672,4.839) | 1.616 | (1.601,1.631) | < 0.0001 | 16.761 |
|  | S2 | 2,763,596 | 168,682 | 6.104 | 2,991,170 | 86,606 | 2.895 | 3.208 | (3.174,3.242) | 1.854 | (1.838,1.869) | < 0.0001 | 12.861 |
|  | Female | 1,455,537 | 159,103 | 10.931 | 1,587,602 | 77,001 | 4.85 | 6.081 | (6.02,6.141) | 2.028 | (2.01,2.045) | < 0.0001 | 44.57 |
|  | Male | 991,809 | 67,077 | 6.763 | 1,049,026 | 36,579 | 3.487 | 3.276 | (3.216,3.337) | 1.727 | (1.705,1.749) | < 0.0001 | 25.641 |
|  | Black | 489,246 | 48,810 | 9.977 | 529,627 | 26,072 | 4.923 | 5.054 | (4.952,5.156) | 1.769 | (1.743,1.796) | < 0.0001 | 25.729 |
|  | White | 1,740,901 | 157,290 | 9.035 | 1,865,375 | 86,012 | 4.611 | 4.424 | (4.372,4.476) | 1.906 | (1.89,1.921) | < 0.0001 | 75.883 |
| **Other chronic obstructive pulmonary disease** | Primary | 3,070,834 | 156,956 | 5.111 | 3,114,487 | 83,199 | 2.671 | 2.44 | (2.409,2.47) | 1.7 | (1.686,1.714) | < 0.0001 | 279.048^26^ |
|  | S1 | 1,025,904 | 59,797 | 5.829 | 1,025,532 | 45,725 | 4.459 | 1.37 | (1.31,1.43) | 1.244 | (1.229,1.259) | < 0.0001 | 0.033 |
|  | S2 | 3,043,316 | 99,483 | 3.269 | 3,094,534 | 53,092 | 1.716 | 1.553 | (1.529,1.578) | 1.657 | (1.639,1.674) | < 0.0001 | 41.717 |
|  | Female | 1,640,439 | 82,878 | 5.052 | 1,662,474 | 39,001 | 2.346 | 2.706 | (2.666,2.747) | 1.885 | (1.863,1.908) | < 0.0001 | 166.376 |
|  | Male | 1,051,178 | 54,887 | 5.221 | 1,066,750 | 30,335 | 2.844 | 2.378 | (2.325,2.431) | 1.63 | (1.607,1.653) | < 0.0001 | 74.904 |
|  | Black | 556,972 | 26,389 | 4.738 | 562,337 | 14,249 | 2.534 | 2.204 | (2.135,2.273) | 1.578 | (1.546,1.61) | < 0.0001 | 7.276 |
|  | White | 1,886,730 | 106,488 | 5.644 | 1,915,969 | 59,248 | 3.092 | 2.552 | (2.511,2.593) | 1.754 | (1.736,1.772) | < 0.0001 | 253.471 |

**Supplement Table 2.** Impact of overweight and obesity on chronic inflammatory disease (CID) manifestation. Primary, S1, sex- and race-stratified analyses considered outcomes with no restriction to follow up. In sensitivity analysis S2 outcomes occurring three months to five years after the index event were consider. For all analyses, except sensitivity analysis S1, the endpoint was obesity and overweight (ICD10:E66). For sensitivity analysis S1, all EHRs were also retrieved from those with documented ICD10:Z00. Within this cohort, a BMI (LOINC 391 56-6) >30 kg/m^2^ defined the overweight/obese group, and a BMI ranging from 18.5 to 25.0 kg/m^2^ defined the non-obese/overweight group. ^1-26^Hazard ratios and its associated confidence intervals, together with the test for proportionality is calculated using R's Survival package v3.2-3. The larger the Chi-square is, the lower is the proportionality. Therefore, we here report odds ratio (OR), 95% confidence intervals an p value if Chi square is 10 or higher for the primary analysis. ^1^OR 3.338 (3.317,3.359) p<0.0001, ^2^OR 1.871 (1.863,1.879) p<0.0001, ^3^OR 4.558 (4.43,4.69) p<0.0001, ^4^OR 1.903 (1.854,1.954) p<0.0001, ^5^OR 1.765 (1.711,1.822) p<0.0001, ^6^OR 1.691 (1.666,1.717) p<0.0001, ^7^OR 2.022 (1.96,2.087) p<0.0001, ^8^OR 1.985 (1.965,2.006) p<0.0001, ^9^OR 1.866 (1.811,1.923) p<0.0001, ^10^OR 1.93 (1.901,1.958) p<0.0001, ^11^OR 1.898 (1.821,1.979) p<0.0001, ^12^OR 1.987 (1.917,2.054) p<0.0001, ^13^OR 1.801 (1.701,1.906) p<0.0001, ^14^OR 1.412 (1.371,1.454) p<0.0001, ^15^OR 1.314 (1.278,1.349) p<0.0001, ^16^OR 1.552 (1.512,1.593) p<0.0001, ^17^OR 1.467 (1.436,1.499) p<0.0001, ^18^OR 1.871 (1.863,1.879) p<0.0001, ^19^OR 1.732 (1.67,1.793) p<0.0001, ^20^OR 1.573 (1.506,1.643) p<0.0001, ^21^OR 2.45 (2.366,2.538) p<0.0001, ^22^OR 4.071 (4.005,4.139) p<0.0001, ^23^OR 1.681 (1.67,1.693) p<0.0001, ^24^OR 1.876 (1.866,1.886) p<0.0001, ^25^OR 2.201 (2.185,2.216) p<0.0001, ^26^OR 1.963 (1.946,1.979) p<0.0001. *Outcomes had not been run during the initial analysis. Data for these outcomes was analyzed on July 6^th^, 2024. Therefore, the sample size is greater than indicated in Supplement Table 1.
